# Evidence for Diffuse Central Retinal Edema *In Vivo* in Diabetic Male Sprague Dawley Rats

**DOI:** 10.1371/journal.pone.0029619

**Published:** 2012-01-11

**Authors:** Bruce A. Berkowitz, David Bissig, Yongquan Ye, Puja Valsadia, Timothy S. Kern, Robin Roberts

**Affiliations:** 1 Department of Anatomy and Cell Biology, Wayne State University School of Medicine, Detroit, Michigan, United States of America; 2 Department of Ophthalmology, Wayne State University School of Medicine, Detroit, Michigan, United States of America; 3 Department of Radiology, Wayne State University School of Medicine, Detroit, Michigan, United States of America; 4 Department of Medicine, Case Western Reserve University and Veterans Administration Medical Center Research Service 151, Cleveland, Ohio, United States of America; University of Florida, United States of America

## Abstract

**Background:**

Investigations into the mechanism of diffuse retinal edema in diabetic subjects have been limited by a lack of animal models and techniques that co-localized retinal thickness and hydration *in vivo*. In this study we test the hypothesis that a previously reported supernormal central retinal thickness on MRI measured in experimental diabetic retinopathy *in vivo* represents a persistent and diffuse edema.

**Methodology/Principal Findings:**

In diabetic and age-matched control rats, and in rats experiencing dilutional hyponatremia (as a positive edema control), whole central retinal thickness, intraretinal water content and apparent diffusion coefficients (ADC, ‘water mobility’) were measured *in vivo* using quantitative MRI methods. Glycated hemoglobin and retinal thickness *ex vivo* (histology) were also measured in control and diabetic groups. In the dilutional hyponatremia model, central retinal thickness and water content were supernormal by quantitative MRI, and intraretinal water mobility profiles changed in a manner consistent with intracellular edema. Groups of diabetic (2, 3, 4, 6, and 9 mo of diabetes), and age-matched controls were then investigated with MRI and all diabetic rats showed supernormal whole central retinal thickness. In a separate study in 4 mo diabetic rats (and controls), MRI retinal thickness and water content metrics were significantly greater than normal, and ADC was subnormal in the outer retina; the increase in retinal thickness was not detected histologically on sections of fixed and dehydrated retinas from these rats.

**Conclusions/Significance:**

Diabetic male Sprague Dawley rats demonstrate a persistent and diffuse retinal edema *in vivo*, providing, for the first time, an important model for investigating its pathogenesis and treatment. These studies also validate MRI as a powerful approach for investigating mechanisms of diabetic retinal edema in future experimental and clinical investigations.

## Introduction

There are few non-invasive treatment options for diffuse retinal edema (DRE) in patients with diabetes. DRE is associated with vision loss and poor prognosis following laser treatment [1]–[3]. In this study, we define experimental DRE as an abnormal buildup of fluid in the retina that results in wide-spread and prolonged supernormal thickness. This buildup can occur when normal homeostasis between fluid influx and efflux is altered [1], [4], [5].

Study and treatment of DRE have been limited by the lack of both relevant diabetic animal models and analytical *in vivo* biomarkers of DRE [6]. Commonly used metrics for retinal edema include retinal morphology/thickness (e.g., optical coherence tomography or histology), BRB integrity (e.g., leakage and accumulation of exogenous biomarkers, or damage to tight junction proteins), or the extent of isolated Muller cell swelling in response to an osmotic challenge [6]–[9]. However, such assessments are generally incomplete since these biomarkers do not spatially co-localize changes in intraretinal thickness and water content in the same eye *in vivo*.

Previously, we reported a significant central retinal thickening on MRI in 3 mo diabetic male Sprague Dawley (SD) rats *in vivo*; passive blood-retinal barrier damage was evident only at much longer durations of diabetes [9]–[11]. Studies with albumin-associated tracers report damage to BRB prior to 3 mo of diabetes in male SD rats [8], [12]. However, it remains unclear whether or not such early retinal thickening and/or loss of transcellular BRB integrity represent edema (as defined above) in male SD rats.

In this study, we address this question by first determining if central retinal thickening persisted over time in diabetic male SD rats. Then, to determine if the thickening seen on MRI represented fluid buildup, we developed and applied MRI methods that specifically measured retinal thickness, water content, and water mobility (i.e., apparent diffusion coefficient (ADC)) in co-localized central regions *in vivo*. ADC is a function of the number and separation of, and movement through cellular barriers, and is a sensitive biomarker of edema [13]. These methods were applied in both a dilutional hyponateria (DH) model (positive control) and in 4 mo diabetic male SD rats.

## Results

### Systemic physiology

Body weight and glycated hemoglobin values are summarized in Table 1. In Arm 1 both parameters increased with age (linear regression analysis, r = 0.76; P<0.05, r = 0.5, P<0.05, respectively). In Arm 2, the three sets of controls had similar (one-way ANOVA; P>0.05) weights (Table 1). Similar A1C values were found between the two arms (P>0.05).

**Table 1:** Summary of Body Weight and Glycated Hemoglobin (A1C).

Groups	Body Weight (g)	A1C (%)
***Arm A***		
C	493±8	5.66±0.07
2 mo D	377±14	12.37±0.35
		
C	480±22	6.17±0.1
3 mo D	387±5	14.59±0.49
		
C	582±16	6.62±0.08
4 mo D	414±6	12.23±0.29
		
C	587±12	6.00±0.17
6 mo D	461±12	13.40±0.56
		
C	645±18	6.62±0.13
9 mo D	456±15	14.69±0.48
		
***Arm B***		
C	501±10	5.75+0.10
		
preDH	496±33	–
		
D (4 mo)	369+11	13.07+0.47

### Arm 1

#### Time course of retinal thickness measured via MRI

**Figure 1:**
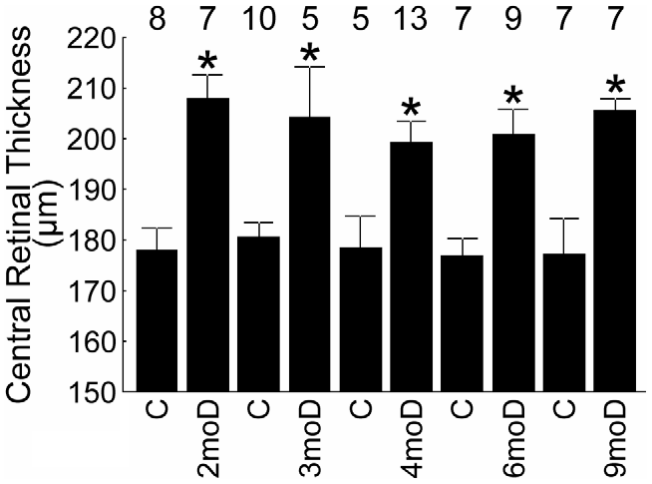
Summary of central retinal thickness. Data are presented for the diabetic (D, diabetes duration given) and age-matched controls (C) male SD groups. These results strongly support our previous observations of diabetes-induced thickening in this model *in vivo* [10]. *, significant compared to age-matched control, *P*<0.05. Error bars represent SEM, and numbers above the bars represent number of animals studied.

At each time point (i.e., 2, 3, 4, 6, and 9 mo of diabetes), diabetic male SD rats had a total retinal thickness that was greater (P<0.05) than that of age-matched controls (Figure 1); within the diabetic and control groups, no change (P>0.05) in thickness with age was noted. No differences (P>0.05) in retinal thickness based on histologic sections were noted between a subset of 9 mo diabetic rats and age-matched controls (data not shown, 4 mo diabetic and control data presented below).

### Arm 2

#### Retinal thickness and water content

**Figure 2:**
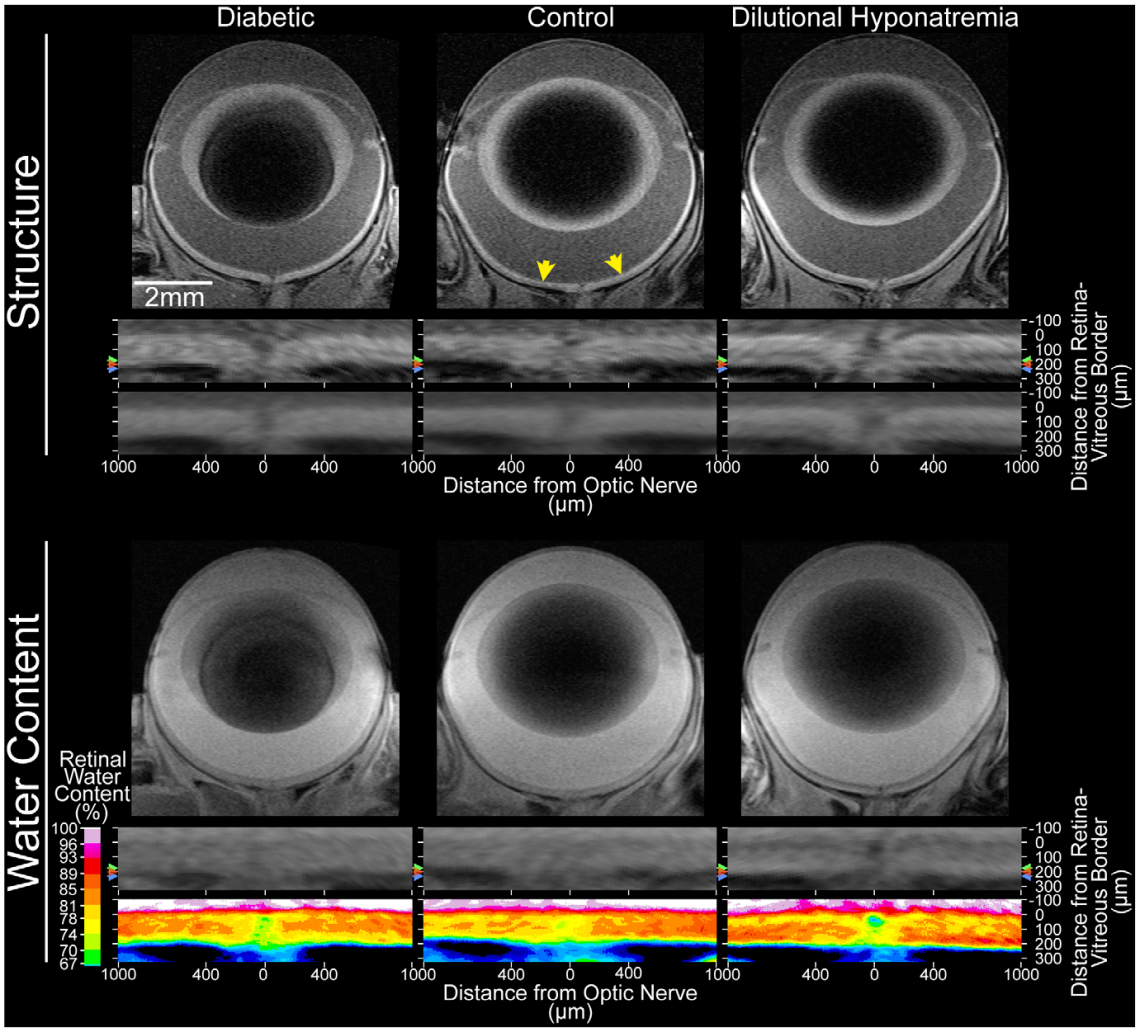
Representative structural and water content images. Structural (*top*; unmodified spin-echo) and water content (*bottom*; turbo-FLASH) images from a diabetic rat (left column), and a second rat both before (control; middle column) and after (right column) dilutional hyponatremia. *Top*: Directly under each image, the central retina (±1 mm from the optic nerve head; yellow arrows in top center) is shown after linearization. Colored arrows to the left and right of those animals' linearized retinas indicate the location of the retina/choroid border for each; red – diabetic, blue – dilutional hyponatremia, green – control (before dilutional hyponatremia). Those arrows also indicate the outer border used for the central retinal thickness measurement, since the linearized retinas are shown after co-alignment of the vitreoretinal border. Consistent with the appearance of the structural images, the diabetic and dilutional hyponatremia retinas are visibly thicker than the control retina. The same pattern is visible in group average images, which are shown below each representative subject's linearized retina. The reduced clarity of the retina/choroid border in group average images is due to inter-individual differences in retinal thickness: Since linearized retinas are co-aligned to the vitreoretinal border in this figure, group average images (without spatial normalization) will average retina from one subject with non-retina of another at the retina/choroid border due to the range of retinal thicknesses in each group (diabetic: 199–244 µm; control: 149–203 µm; dilutional hyponatremia 198–227 µm). Such considerations motivated our resampling of retinal profiles onto a %thickness (rather than µm) scale prior to formal analysis (e.g. Figs. 3 and 5). *Bottom*: As above, the group average is presented directly beneath the linearized central retina for representative subjects. Here, though, the color-maps shows %water content (%v/v) values after setting vitreous to 99% (see 3^rd^ paragraph of the ‘MRI Image Analysis’ section within Methods). Note that both retinal thickness and %water content will determine the total water content of a retina.

**Figure 3:**
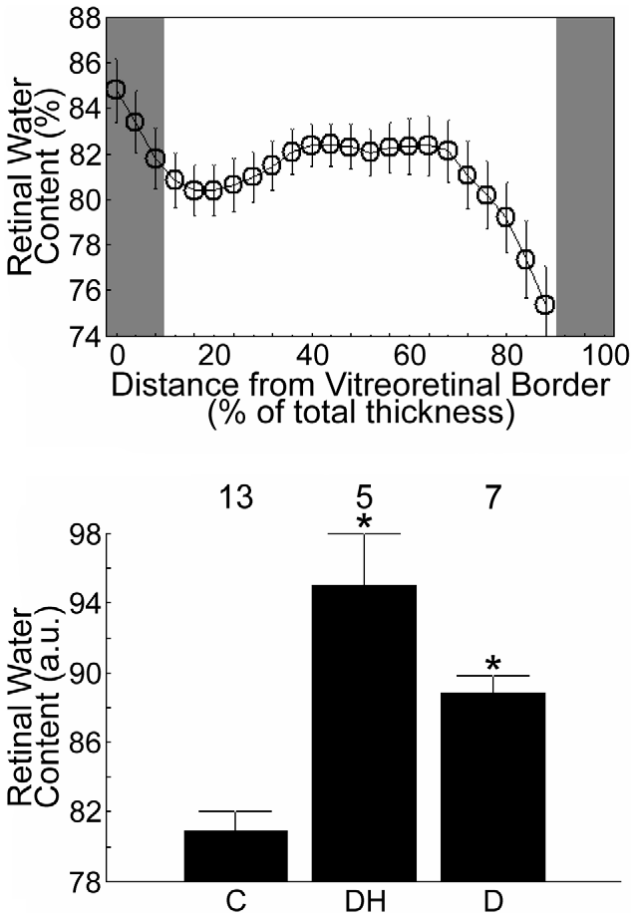
Summary of retinal water content. *Top*: Absolute intraretinal water content across the central retina in control rats. The y-axis scaling in this Figure was set to help visualize the variations in intraretinal water content. With this scaling, values <74% are not shown. Due to partial volume averaging and the non-linear effects of the very fast flow in the choroidal circulation (relative to that in the inner retina), water content estimates at depths beyond 88% are considered inaccurate. This region, as well as the tissue near the vitreoretinal border (at 0% thickness), are gray-shaded to indicate that they are excluded from further analysis (see Appendix S1 for more detail). Error bars represent SEM. *Bottom*: Grand means of relative (see methods) central intraretinal water content (i.e., from 12–88% thickness) for control rats (C & preDH data, here, “C”), dilutional hyponatremia group (postDH data, here, “DH”), and the diabetic group (D). In both graphs, error bars represent SEM. Numbers above the bars represent number of animals studied.

In the control rats for this Arm, total retinal thickness measured via MRI was 182±4 µm (mean ± SEM, n = 17), which was not different (P>0.05) from age-matched controls in Arm 1 (178±6 µm, n = 5). Intraretinal water content is illustrated in Figure 2 and summarized in Figure 3. The control group had an overall (i.e., from 12 through 88% depth) absolute water content of 80.9±1.1% (n = 13). We confirmed the linearity of MRI signal intensities with proton content in H_2_O : D_2_O phantom studies (r = 0.96, P<0.05, data not shown).

In the DH group, postDH total retinal thickness (227±8 µm; *n* = 5) was significantly greater (P<0.05) than that of the combined control group (see above) as well as the preDH subset of controls (mean postDH – preDH = 54±8 µm; *n* = 5). Overall water content was greater (P<0.05) than in controls (Figures 2 and 3).

In the 4 mo diabetic rats (the only duration of diabetes studied in Arm 2), total retinal thickness (211±4 µm, n = 7) was significantly greater (P<0.05) than that of the combined controls (see above). Histologically, in diabetic rats, central retinal thickness (158±6 µm, n = 5) was not different from their age-matched control values (146±6 µm, n = 6); both groups demonstrated the expected shrinkage upon processing relative to data *in vivo* (data not shown) [14]. Diabetic retinal hydration on MRI was supernormal (P<0.05, Figures 2 and 3), but not different (P>0.05) from that in postDH rats.

Note that an assumption of our MRI retinal hydration measure is that the thicker retina contained as much water per unit volume as in the control central retina (i.e., 80.9%). However, similar statistical conclusions for both experimental groups (i.e., postDH and D) are achieved if much lower amounts of water (≥65%) are used in the calculation of total retinal water content (data not shown).

#### Intraretinal water mobility (ADC)

**Figure 4:**
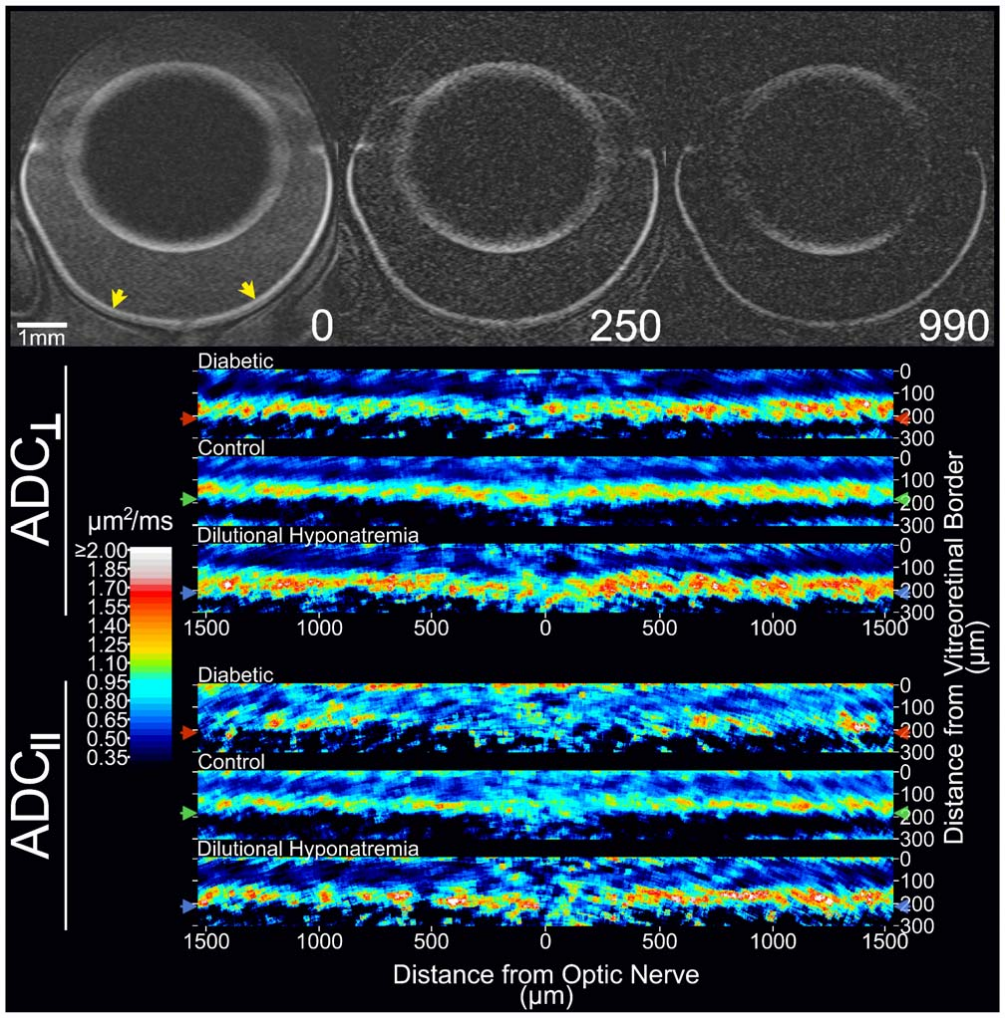
Representative ADC images. *Top (grayscale images)*: Representative images from a control rat (the same as featured in Figure 2) with diffusion weighting applied parallel to the optic nerve (top/bottom of page in this image orientation). These representative images were collected at three different b-values (from left to right, 0, 250, and 990 s/mm^2^). Note that, while signal is lost with mild diffusion weighting in vitreous and anterior chamber – signal there being just above background levels at 250 s/mm^2^ – the retina, with more restricted diffusion, retains signal through b = 990 s/mm^2^. Preferential signal loss in lateral (relative to the anterior and posterior) lens cortex at higher b-values is due to the preferential movement of water parallel to the lens surface. Yellow arrows in the b = 0 image indicate the region of retina linearized and shown below (measuring from the optic nerve head, ∼±30% of the hemiretinal extent). *Bottom (color images)*: Average ADC maps from each group are presented for visualization purposes only since spatial normalization had not yet been applied (see Legend of Figure 2 for additional discussion). Arrows to the left and right of the color maps indicate the group average location of the retina/choroid border. We also note formal qualitative analysis of ADC (Figure 5) was performed on the linearized and group averaged retinal profile.

**Figure 5:**
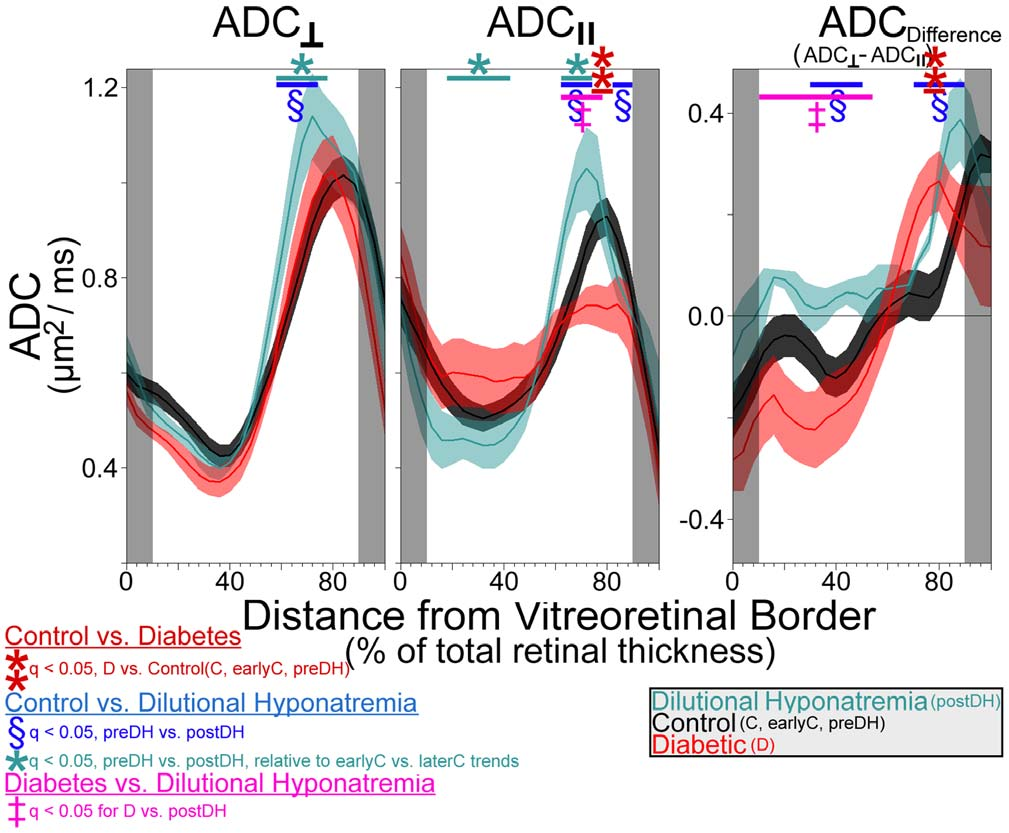
Group comparisons of intraretinal ADC_‖‖_ and ADC_⊥_ (i.e., water mobility). Water mobility profiles for control (black; average of groups C, earlyC, and and preDH; n = 17), dilutional hyponatremia (turquoise; ‘postDH’ group; n = 5), and diabetic (red; group ‘D’; n = 7) retinas. Data points in gray-shaded areas near the vitreoretinal border (0% thickness) and retina/choroid border (100% thickness) are excluded to minimize partial volume averaging with non-retinal tissue (see Appendix S1 for more detail). The solid lines and shaded areas of profiles represent mean and SEM's, respectively. Note that ADC data from the DH group are plotted with the group average control data (groups C, earlyC, and preDH), but statistical findings are based on paired comparisons: Locations where differences between preDH and postDH significantly greater than zero are shown, as are locations where preDH-to-postDH changes were statistically different than the (non-significant) earlyC-to-laterC changes. In either case, we find a significant, heterogeneous, influence of DH on retinal ADC.

In the non-diabetic controls, visual inspection of the ADC profiles suggested that water movement was most restricted midway through the thickness of the retina (∼32–44% thickness) in both parallel (ADC_‖‖_) and perpendicular (ADC_⊥_) directions relative to the optic nerve, compared with more anterior or posterior retinal regions (Figure 4); statistical analysis supported this impression (Figure 5, P<0.05). Water mobility in the parallel direction was greater than in the perpendicular direction midway through the retina (i.e., ADC_‖‖_>ADC_⊥_; q<0.05 from 36–44% thickness; see Appendix S1 and Figures 4 and 5). A reversal of that pattern (i.e. ADC_‖‖_<ADC_⊥_) was noted near the retina/choroid border (q<0.05 starting at 84% thickness).

In the DH group, both ADC_‖‖_ and ADC_⊥_ were supernormal in the outer retina (q<0.05; including 64–72% thickness). However, ADC_‖‖_ was significantly reduced in the nominal inner retina (q<0.05 from 32–48% thickness, Figures 4 and 5) and in a small section of putative far-outer retina (76–80% thickness). Like in controls, ADC_⊥_ was higher than ADC_‖‖_ in the nominal outer half of the postDH retinas (q<0.05 from 68–88% thickness). However, postDH retinas lacked the characteristic mid-retinal section where ADC_‖‖_>ADC_⊥_ (ADC_⊥_ trends higher than ADC_‖‖_ throughout the putative inner retina, reaching q<0.05 at 16–20%). The difference between ADC_⊥_ and ADC_‖‖_ was significantly exaggerated (higher, relative to preDH controls) in the nominal outer half (q<0.05 from 76–88% thickness) of postDH retinas, but diminished in the apparent inner half (i.e. closer to 0; q<0.05 from 32–48% thickness). In the diabetic rats, ADC_‖‖_ was subnormal in the putative outer half of the retina (Figures 4 and 5; q<0.05; 76–80% thickness) relative to controls. Like in controls, ADC_⊥_ was higher than ADC_‖‖_ in portions of the nominal outer retina (q<0.05; 72–88%) near the retina/choroid border. As with postDH retinas, this difference between ADC_⊥_ and ADC_‖‖_ was significantly exaggerated (higher than in controls) in the apparent outer retina (q<0.05; 76–80% thickness). Unlike the postDH retinas, a somewhat normal apparent inner retinal pattern of ADC_‖‖_>ADC_⊥_ was also present in diabetic retinas with ADC_‖‖_ appearing greater than ADC_⊥_ over a larger-than-normal expanse of retina (q<0.05; 12% and at 20–48%) although the difference did not reach statistical significance (q>0.05) relative to controls, it did differ significantly from the postDH retina (q<0.05 at 12–52%). These results are consistent with the presence of edema in the diabetic retina but also suggest that diabetes induces changes in intraretinal water handling that are somewhat complex relative to that in DH (see below).

## Discussion

In this study, we 1) confirmed a supernormal central retinal thickness diabetic male SD rats via MRI and extended these results to 2–9 mo of hyperglycemia, 2) validated (in a DH retinal edema model) the sensitivity and physiologic accuracy of new MRI methods for assessing central retinal water content and mobility [15], and 3) found first-time evidence that central retinal thickening in rats following 4 mo of diabetes is due to edema. Currently, it is not clear if only diabetic male SD rats demonstrate DRE. We previously reported that the retinas of 3 mo diabetic female SD or female Lewis rats, as well as diabetic male C57Bl/6 mice, were not thicker than their respective controls on MRI examination suggesting a lack of edema [10], [15], [16]. Additional studies are needed to better understand why retinal thickening/edema develops in some, but not all, models.

The reported differences in male SD rat retinal thickness are based on a total of 59 controls and 56 diabetic rats (combining the data in this study and our previous report [10]). All of these results were generated over several years using many different batches of rats and studied with different image sequences on two different MRI systems. Such experimental variation would be expected to conspire against finding any differences if they were the result of noise or group bias. Furthermore, recent *in vivo* OCT data support the noted retinal thickness differences [17]. It is possible that subtle changes in retinal thickness occurred over time that were below our temporal and/or detection sensitivity limits although in a previous study in the C57Bl/6 mice we had no problem detecting retinal thinning in controls [16], [18]. These considerations speak to the accuracy of the MRI thickness measure and robustness of the difference when measured *in vivo*.

In nondiabetic male SD rats, the accuracy of the MRI retinal thickness measurement has been validated against histology [19] and OCT results [17]. These control values appear stable over time since postnatal day 50 male SD rats had a thickness (187±6 µm, n = 5, from an unpublished study) that was similar to the older controls in this study (P>0.05). We also found herein that systemic manganese (Arm 1) did not alter retinal thickness, compared to that measured in Arm 2. Since the MRI measurement of retinal thickness agreed with standard histologic preparations in nondiabetic animals, we were somewhat surprised to find that MRI and histology produced different results regarding retinal thickening in diabetic animals. In early stage diabetes, male SD rats have been reported to have supernormal retinal thickness on MRI and OCT [15], [17], whereas measurements of retinal thickness by histologic methods have yielded inconsistent results with thicknesses that are reported to be either unchanged (this study) [20], decreased [21]–[26], or increased [ 25], [27]. *Ex vivo* histologic measurements might be confounded by effects of sample preparation (e.g., dehydration) and processing that are inherent in histology and which might vary between labs. It is worth noting that MRI and OCT provide complementary information about central retinal thickness. In the context of retina edema in experimental diabetes, compared to OCT, the use of MRI is advantageous because it: a) is not sensitive to media opacities such as cataracts allowing for the impact of greater durations of diabetes and longer treatment regiments on the retina to be studied, and b) can provide co-localized measures of key edemagenic (retinal thickness, blood retinal barrier breakdown, and water content and mobility) and functional (MEMRI) parameters in the same eye panretinally (i.e., ora serrata-to-ora serrata).

Few methods are available for evaluating absolute retinal water content *in vivo*. In this study, retinal hydration (e.g., 80.9±1.1% in control SD rats) was derived by assuming a pre-retinal vitreous water content of 99% [28]. Although reasonable, the fidelity of this assumption in the experimental groups remains to be ascertained. If necessary, future studies could utilize an external water standard as an alternative reference. Note that the degree of retinal hydration measured in controls was similar to a previous estimate of water content (84%, range 82–86%) obtained *ex vivo* by measuring the specific gravity of retinas from female SD rats [6]. The influence of the rat's sex on retinal water content has not been studied and may be a somewhat confounding factor in the comparison of the present results and those in the literature. In addition, the specific gravity values were obtained from excised retinas and could have overestimated the amount of retinal water due to, for example, incomplete retinal removal (i.e., not excising sub-retinal regions which appear to contain less water than the rest of the retina; see Figure 2), and/or increased tissue protein levels following sacrifice [29].

Edema can form when the balance between retinal water influx and efflux is altered. DH was used in this study as a positive control condition since it increases retinal thickness in the absence of BRB breakdown, and, in the brain, increases hydration and reduces ADC, relative to controls [15], [30], [31]. The findings of the present study are consistent with these brain results since DH increased water content and reduced mobility (in the parallel direction) in the nominal inner retina. Subnormal mobility is thought to arise when water accumulates inside the cell (i.e., a small space with restricted motion) resulting in cell swelling (or cytotoxic edema). The supernormal ADC found *in both directions* in the putative outer retina may reflect osmotically-induced changes in the shape of rods and/or Muller cells, such as swelling to a more spherical shape [32]. The small section of subnormal ADC_‖‖_ in the far outer retina of DH rats may reflect specific swelling of rod outer segments causing a decrease in the extracellular volume between rods [33]. In any event, these DH experiments allowed us to validate the usefulness of MRI to measure co-localized retinal thickness, hydration, and water mobility linked with edema.

Caution is warranted when trying to compare the DH and diabetes data since the DH results represent a single time point in the evolving response of normal retinas (i.e., retinas with functional fluid handling capacity) acutely exposed to a hypoosmotic challenge. In contrast, diabetes represents a chronic condition with potential long term changes in retinal fluid handling [7]. We found that normally water movement in the mid-retina is relatively less restricted in the parallel than perpendicular directions relative to the optic nerve, and that this difference was exaggerated in diabetic retinas, but diminished in DH retinas. Greater diffusion in the parallel than perpendicular direction suggests a bias for movement through elongated structures parallel to the optic nerve.

Muller cells obtained from diabetic rat retinas do not handle osmotic stress normally and swell in response to a hypotonic environment [7], [34]. Diabetes has also been reported to reduce RPE sodium potassium ATPase activity and, theoretically at least, increase outer retinal hydration [35], [36]. Visually, the intraretinal ADC profile is clearly different between D and C or DH groups. Significant differences between diabetic and control ADCs were restricted to the far outer half of the retina (76–80% thickness), which is consistent with the location of the rod outer segments and extracellular fluid found in the subretinal space. As with the DH group, this far outer retinal (≥76% thickness) decrease in ADC_‖‖_ may reflect narrowing of the extracellular space between outer segments. The present finding that the intraretinal ADC profile of diabetics differs from that in controls justifies future work to fully characterize the retinal diffusion abnormalities associated with diabetic retinal edema.

In the present work, the mechanism of retinal edema (i.e., cytotoxic (intracellular edema in the absence of BRB damage) or vasogenic (extracellular edema with BRB breakdown) edema) in the diabetic rats is somewhat unclear. In retinal tissue, water is mostly (90–95%) intracellular [37]. However, MRI water content measurements probably do not reflect hydration levels of the intracellular compartment *per se* since water resides in each compartment for short times (∼msec) relative to the time it takes to acquire an image (∼minutes) [38]. In other words, water movement between compartments is expected to cause some degree of blurring of intra- and extra-cellular hydration information preventing a simple water-compartment-specific interpretation of the present data.

ADC measurements are relevant to the present investigation because they provide an additional edema-sensitive MRI biomarker [13]. Changes in water mobility reflect microstructural alterations and thus change as a consequence of edema. Increases in ADC are often observed in models of vasogenic edema presumably because more water in the extracellular space is less restricted; decreases in ADC typically found following an osmotic provocation as a likely consequence of more intracellular water in a more restricted space [39]. In the present study, subnormal ADCs are putatively considered representative of cytotoxic edema. However, ADC patterns differ between diabetic and DH groups. Insofar as DH models ‘pure’ cytotoxic edema – uniform cell swelling without BRB compromise – these data argue against diabetic retinal edema as being only or simply cytotoxic. Alternatively, cytotoxic and vasogenic influences on ADC may be convoluted in the diabetic case. Until further investigations can be performed to examine more closely how intraretinal ADC changes in diabetic rats, we conservatively interpret the diffusion data as demonstrating the presence of edema but reserve judgment as to how to interpret the changes in terms of specific mechanisms.

A common biomarker of vasogenic edema is loss of BRB integrity. In male SD rats diabetic for 4 mo, we find no increase in paracellular (i.e., passive) movement of tracer in the retina [9], [10]. In contrast, in diabetic male SD rats, increased transcellular BRB damage has been reported using, for example, albumin-associated tracers [8], [12]. Although the physiology underlying these albumin-associated tracer studies is unclear, such BRB data support, at least provisionally, a vasogenic edema in diabetic male SD rats [9], [40]. In any event, measuring only the degree of BRB damage, without assessing co-localized retinal thickness and hydration *in vivo*, is clearly insufficient as a measure for edema [5], [6].

In conclusion, MRI methods have been developed, validated, and applied to measure key edemagenic-factors (retinal thickness, water content and mobility) *in vivo*. All three of these parameters are needed to quantify edema and we use these tools to demonstrate, for the first time, the presence of a DRE in male SD rats. In addition, the present studies underscore the value of ADC in investigating DRE *in vivo*. We envision future MRI studies to investigate mechanisms and treatments for DRE in longitudinal preclinical and clinical investigations.

## Materials and Methods

 Animals were treated in accordance with the Principles of Laboratory Animal Care (National Institutes of Health publication no. 85−23, revised 1985; http://grants1.nih.gov/grants/olaw/references/phspol.htm) and with the ARVO Statement for the Use of Animals in Ophthalmic and Vision Research. All rats were housed and maintained in normal 12-hour cycled laboratory lighting, unless otherwise noted. This study received full approval (approval ID A060808) by the Wayne State University School of Medicine Institutional Animal and Care Use Committee (IACUC).

### Animal Groups

Male SD rats were examined in both arms of this study, after either being made diabetic when 2 mo. old, or retained as non-diabetic age-matched controls. In the first arm, we measured central retinal thickness at 2, 3, 4, 6, and 9 mo after induction of diabetes, and in age-matched non-diabetic controls (ages 4, 5, 6, 8, and 10 to 11 months) (see Figure 1 for n's per time point). In the second arm, we selected a single time-point (age: 6 mo) and measured retinal thickness, intraretinal water content, and ADC in the following four groups: (i) Rats experiencing dilutional hyponatremia (DH): After being anesthetized with urethane (in preparation for MRI, see below), non-diabetic rats (n = 5) were scanned (“preDH”), then injected intraperitoneally with distilled water (three injections, 50 ml per kg body weight, spaced five minutes apart [15]) before a second MRI scan (“postDH”; with 3.8±0.2 hrs (mean ± SEM) between the starts of scanning periods), (ii) non-diabetic control rats used to test the reproducibility of ADC measurements throughout the thickness of the retina (n = 4), especially regarding the prolonged anesthesia used in the DH group, were scanned (“earlyC”), then maintained for several hours before a second scan (“laterC”, 4.6±0.3 hrs between starts of scanning periods), (iii) diabetic male SD rats (“D”) were scanned once (n = 7), and (iv) a corresponding set of age-matched controls (“C”) were scanned once (n = 8).

Diabetes was induced with intraperitoneal injection of streptozocin (60 mg/kg) within 5 minutes of its preparation in 0.01 M citrate buffer (pH 4.5) in rats with body weights of approximately 200 g after overnight fast. Diabetes was verified 3 days later by the presence of plasma hyperglycemia (≥300 mg/dl) and elevated urine volume (more than 60 ml/d) in nonfasted rats. Rat body weight and blood glucose levels were monitored weekly. Subtherapeutic levels of insulin (0–2 U neutral protamine Hagedorn insulin administered subcutaneously daily) were administered to maintain blood glucose levels between 450 and 550 mg/dl without urine ketones. Glycated hemoglobin was measured after 2 months of diabetes (Glyco-Tek affinity columns, kit 5351; Helena Laboratories, Beaumont, TX).

### MRI data acquisition

Rats were maintained in darkness overnight and at all times the next day. Immediately before MRI, rats were anesthetized using urethane (36% solution administered intraperitoneally, 0.083 ml/20 g animal weight, prepared fresh daily; Sigma-Aldrich, Milwaukee, WI). MRI data were acquired on 4.7 T or 7 T MRI systems (Bruker Avance and Clinscan, respectively) using a surface coil (1.0 cm diameter) placed over the left eye. In all studies, we collected images from the ocular median (between center of lens and optic nerve head spanning superior to inferior). For Arm 1, we duplicated the conditions of the initial study in which we measured increased retinal thickness in rats that had been treated with manganese chloride (to evaluate retinal function) [10]. 2, 6, and 9 mo diabetic (and age-matched control) data were collected at 4.7T, and the 3 and 4 mo diabetic (and respective control) data were acquired at 7T. All procedures (e.g., weighing, injecting MnCl_2_, anesthetic administration, and MRI examination) were conducted under dim red light or darkness. MnCl_2_ was administered as intraperitoneal injection (44 mg/kg) on the right side of awake rats. After this injection, rats were maintained in dark conditions for another 3.5 to 4 hours. On the 4.7 T system, high-resolution images were acquired using an adiabatic spin-echo imaging sequence (repetition time [TR] 350 s, echo time [TE] 16.7 ms, number of acquisitions [NA] 16, matrix size 512×512, field of view [FOV] 12×12 mm^2^, slice thickness 620 µm) [41]. On the 7T system, partial saturation T_1_ data were acquired (TR (see below), TE 13, NA (see below), matrix size 160×320, FOV 8×8 mm^2^, slice thickness 600 µm). At each TR, a number of single images (in parenthesis) were acquired in the following order: TR 0.15 s (6), 3.5 s (1), 1.0 s (2), 1.9 s (1), 0.35 s (4), 2.7 s (1), 0.25 s (5) 0.5 s (3). These acquisition conditions provided 25 µm resolution across central retina. From these data, we measured whole retinal thickness; measures of central intraretinal manganese uptake (a marker of calcium activity/function) were also assessed and will be presented in a separate publication.

For Arm 2, the following acquisition parameters were used to collect 7T data from the same dark adapted eye; no manganese was administered to these rats: (i) *structure*: two spin echo images (TR 1.0 s, TE 13 ms, matrix size 160×320, FOV 8×8 mm^2^, slice thickness 600 µm) were registered (rigid-body) and averaged together for measuring retinal thickness. (ii) *water content*: six 3 d ultra-fast gradient echo images (turbo-FLASH, where the input for “TR” in Siemens' Syngo software (the time between one train of pulses and the following train) was 1000 ms, matrix size 160×320, TE 3.04 ms, FOV 8×8 mm^2^, 16 slices, slice thickness 600 µm, flip angle 3°, and the echo spacing (the time between pulses within a train) was 8.7 ms), were collected such that one of the central slices was co-localized with the structure and diffusion- weighted (described below) images. The six images were registered (rigid-body), averaged together, and used for measuring water content in that central-most slice (see Figure 2 for examples). Note that FLASH sequences with very small flip angles, and in the absence of flow, produce images primarily weighted by water content although T2* might also contribute [42]. We, and others [43], find small differences between vitreous and retinal T2* (data not shown) and thus assume that the present turbo-FLASH images largely reflect vitreous and retina water content. In support of this interpretation, we note that reasonable agreement in retinal water content was found between the present MRI results and previous measures of specific gravity (see below). The turbo-FLASH sequence was also used with a set of phantoms with serial dilution of D_2_O with H_2_O (at room temperature, range 75–99% H_2_O) to evaluate the linearity between known water content and signal intensity (SI). (iii) *water mobility*: diffusion-weighted images (TR 1000 ms, TE 33 ms, matrix size 288×144, FOV 8×8 mm^2^, slice thickness 600 µm, diffusion block duration 4 ms) were acquired with a spin echo sequence modified in-house. The diffusion weighted sequence was developed on the basis of the Siemens product SE (spin echo) sequence by modifying a twice-refocused spin echo diffusion weighting module [44]. One image was collected for each of three directions (slice, read, and phase-encode) at each of four b-values (250, 500, 750, and 990 s/mm^2^), and four images were collected with b = 0. The order in which these images were acquired was randomized for each rat. These images were registered (rigid-body) and analyzed for the apparent diffusion coefficient (ADC). Structure and water mobility scans were performed in each scanning session in Arm 2 (preDH, postDH, earlyC, laterC, D, C), and water content scans were performed in most sessions (preDH, postDH, D, C). In a small subset of rats in control (C, n = 2), distilled water (postDH, n = 1) and diabetic (D, n = 2) groups, we also evaluated blood retinal barrier (BRB) integrity immediately after the aforementioned scanning procedures using dynamic contrast enhanced MRI as previously described; BRB was confirmed to be normal in these subsets of rats from each edema model (data not shown) [9], [10], [15].

In all studies with diabetic rats, at the beginning of the MRI experiment, blood from the tail vein was collected and analyzed for glucose concentration. After the MRI examination, anesthetized animals were humanely killed, eyes enucleated, and the formalin-fixed, paraffin-embedded stained (H&E) sections analyzed by light microscopy. Retinal thickness (including rod outer segments) were measured near the optic disk (both sides) and then averaged.

### MRI Image Analysis

In Arm 1, several images of each eye studied at 7T were available. Where multiple images from a single TR were available, these were averaged after a rigid-body registration step – manually or using the StackReg plugin for ImageJ (Rasband, W.S., ImageJ, U. S. National Institutes of Health, Bethesda, Maryland, USA, http://rsb.info.nih.gov/ij/). The same registration approach was used to co-align images from different TRs for later processing. For Arm 1 animals studied at 4.7T, no registration steps were necessary since only a single image was collected. Next, in-house written software was used to linearize the *in situ* image(s) as previously described [45], [46]. Data from, separately, the superior and inferior central retina (from ±/− 0.4–1.0 mm from the optic nerve) were binned to generate a profile of signal intensity as a function of distance from the vitreoretinal border. For 7T data, profiles were generated for each TR. For each available TR, the previously-validated half-height method ([45]; see Appendix S1 for more detail) was used to identify the retinal borders – the inner border with vitreous, and the outer border with choroid – in the signal intensity profile of each hemiretina [47]. For 4.7T data, these borders were simply subtracted to calculate whole retinal thickness for each hemiretina, and then hemi-retinal thicknesses were averaged to provide a single value for each animal. For 7T data in this Arm, the same approach was applied to each TR, yielding eight values per animal. Although apparent thickness is largely independent of TR, we have found an additional step to be advantageous in previously validated, more heavily-automated, analyses [45] and therefore apply it here: After the mean of all eight values is found, the three values farthest from that retina's mean (highest |z-score|) are ignored. The mean thickness recalculated from the remaining five values is used for any additional steps and analyses.

In Arm 2, where multiple images were available with identical scan parameters (two structural images, six water content images, four images collected at b = 0 using the diffusion sequence) images of each type were registered and averaged as for Arm 1 data. Registration of water content (turbo-FLASH) data to structural data was approached as with between-TR registration in Arm 1. Because of diffusion-dependent signal loss (Figure 4), special consideration was given to registration of diffusion data – particularly images collected with high b-values – to the structural images. After structural (unmodified spin-echo), b0, and diffusion-weighted images were linearized, the half-height method was used to identify the presumptive vitreo-retinal border of each image. A moving-window approach [45] was used to ensure that retinas started the following process aligned to that presumptive border. The next steps in the procedure are detailed in the Appendix S1. Briefly, signal intensity profiles are calculated from the linearized central retina (10–30% hemiretinal extent) [45] at each b-value, and provisional borders are found using variants of the half-height method. Profiles are mutually centered based on these borders, producing highly reliable results throughout the thickness of the retina (see Appendix S1). We note that an alternative analysis using fully-manual registration of diffusion data yielded similar results for comparisons of control with each experimental groups' ADC (not shown). A thorough evaluation of the quality of registration – both in regards to reproducibility of measurements from controls at each increment between 12 and 88%thickness (including comparison of earlyC vs laterC), and in terms of alignment of diffusion features to structural borders, is provided in the Appendix S1.

Intraretinal water content (Arm 2) was measured as follows: First, for each eye, on either side of the optic nerve head, we corrected for the surface coil inhomogeneity using a linear fit to the vitreous SI profile to obtain two preretinal vitreous signal intensities (i.e., within 100 µm from the vitreo-retinal border). The average signal intensity of these two fitted values was adjusted to 99% water content [28]. This adjustment factor was applied to the intraretinal signal intensities from the linearized turbo-FLASH images to yield raw profiles of water content. In control rats, as stated above, the intraretinal water profiles were adjusted in the x-axis direction to be a percentage of total retinal thickness for each animal (with 0% thickness and 100% thickness respectively at the inner and outer borders defined above). In the experimental groups, the same adjustment would not account for any water content change that accompanied increased retinal thickness. Thus, the y-axis was further modified to account for the greater “compression” factor needed for thicker retinas to fit on the 0 to 100% thickness scale. The mean % retinal water content in the increased retinal thickness over that from controls is likely similar to that in the rest of the retina (i.e., 80.9%, see results). In this case, the overall intraretinal mean will not be different between the control and diabetic groups. In other words, examining the mean does not account for any water content change that had to accompany the expanded retinal volume. For this modification, we assume that tissue water concentration in thicker retinas was similar to that found in the control retina (i.e., 80.9% although lower values were later considered; see Results) and that the hydration changes were distributed uniformly across the retina. The latter assumption may not be entirely correct although we had earlier found in diabetic rats that both inner and outer retinal thickness were supernormal [10]. Similar inner and outer retinal thickness measurements were not possible in the present study because the rats were dark adapted making the inner vs. outer boundary difficult to robustly identify. Because these eyes had been scheduled for additional measurements after the MRI experiments (i.e., histology), we were unable to obtain dry weights in these studies. For all of the above reasons, between-group comparisons of water content are presented as an overall water content between 12 and 88% depth into the retina (a range that largely minimized possible partial volume effects at either end of the intraretinal profile).

We provide a detailed account of how water mobility (ADC, Arm 2) was calculated in the Appendix S1. Briefly, after data were linearized and registered, profiles of signal intensity as a function of % depth into the retina were calculated. Between images, signal intensity at b = 0 (S_0_) is highest, and decreases with increasing b-values – more so in tissue with high water mobility (ADC), than with low mobility – sometimes in a direction-dependent fashion. After the four b = 0 images were co-registered and averaged, ADC was calculated using the equation (shown here for the parallel direction):
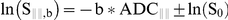
‘S_‖‖,b_’ is the signal intensity with diffusion weighting of a given magnitude (b-value) parallel to the optic nerve, and ‘S_0_’ is the signal intensity without diffusion weighting. Due to the radial symmetry of the eye, the two directions perpendicular to the optic nerve head (i.e., phase-encode and slice) were considered equivalent in the retina and were therefore combined to produce ADC_⊥_ measurements (see Appendix S1); in the central outer retina this metric is perpendicular to the long axis of rod photoreceptors.

### Statistical Analysis

Unless otherwise noted, comparisons – of either paired data, or to age-matched control data – of retinal thickness or intraretinal water content were performed using two-tailed t-tests with P<0.05 considered statistically significant. Since water mobility profiles were compared at several depths into the retina (using 20 statistical tests, from 12% through 88% of the whole retinal thickness in 4% increments), a false discovery rate (FDR) threshold of q = 0.05 was applied, similar to previous work [46], [48]. Unless otherwise noted, statistical comparisons of ADC data used the generalized estimating equation (GEE) approach detailed in the Appendix S1. Data are presented as mean ± SEM.

## Supporting Information

Figure S1 (TIF)

Figure S2 (TIF)

Figure S3 (TIF)

Figure S4 (TIF)

Figure S5 (TIF)

Figure S6 (TIF)

Figure S7 (TIF)

Appendix S1 (DOCX)
