# Supplementary figures and images for: Evidence for Diffuse Central Retinal Edema In Vivo in Diabetic Male Sprague Dawley Rats
Source: PLoS One. 2012 Jan 11;7(1):e29619. doi: 10.1371/journal.pone.0029619 (PMC3256169; doi:10.1371/journal.pone.0029619)

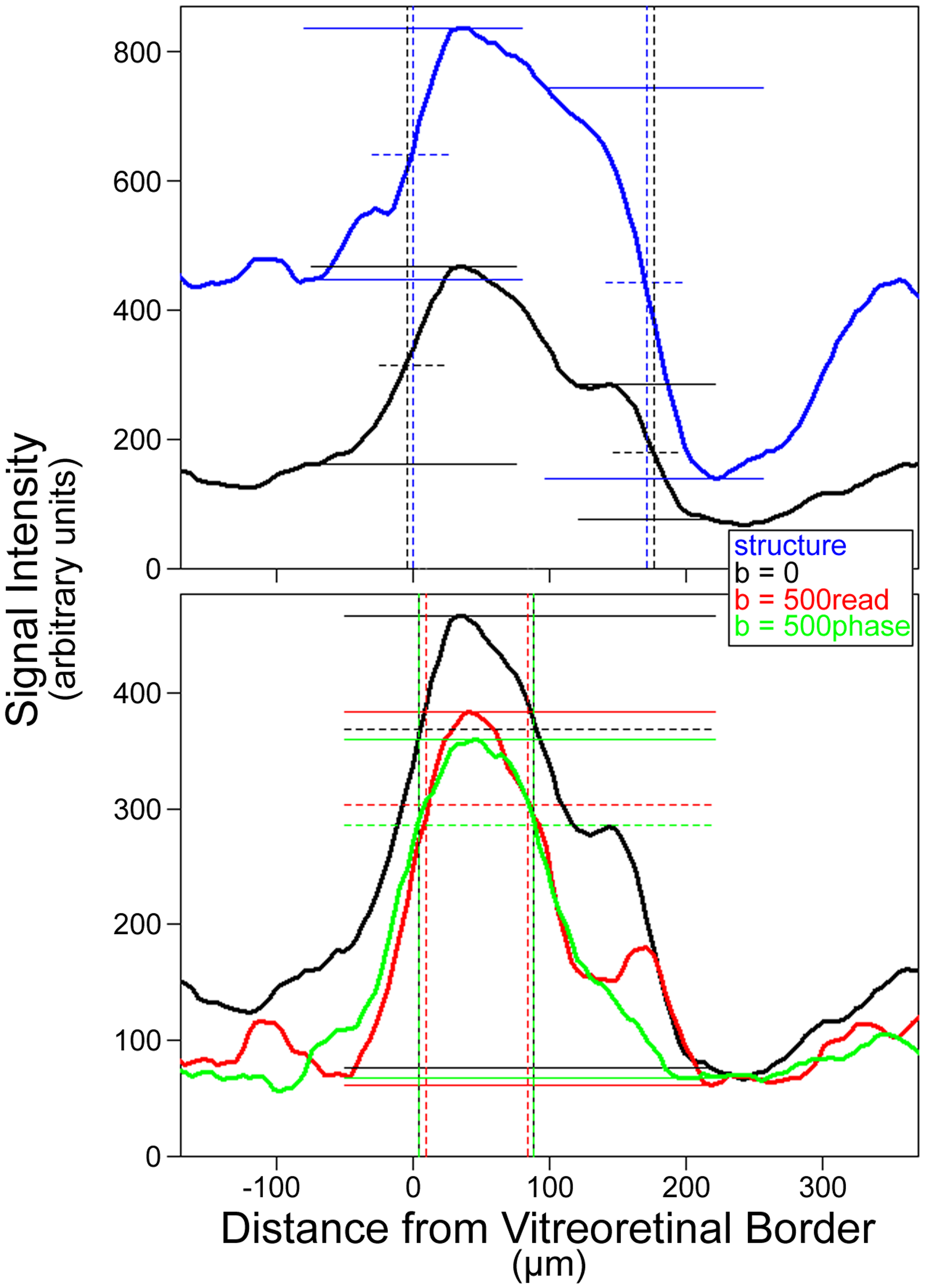

Supplement: Figure S1 — (TIF) [file pone.0029619.s001.tif]

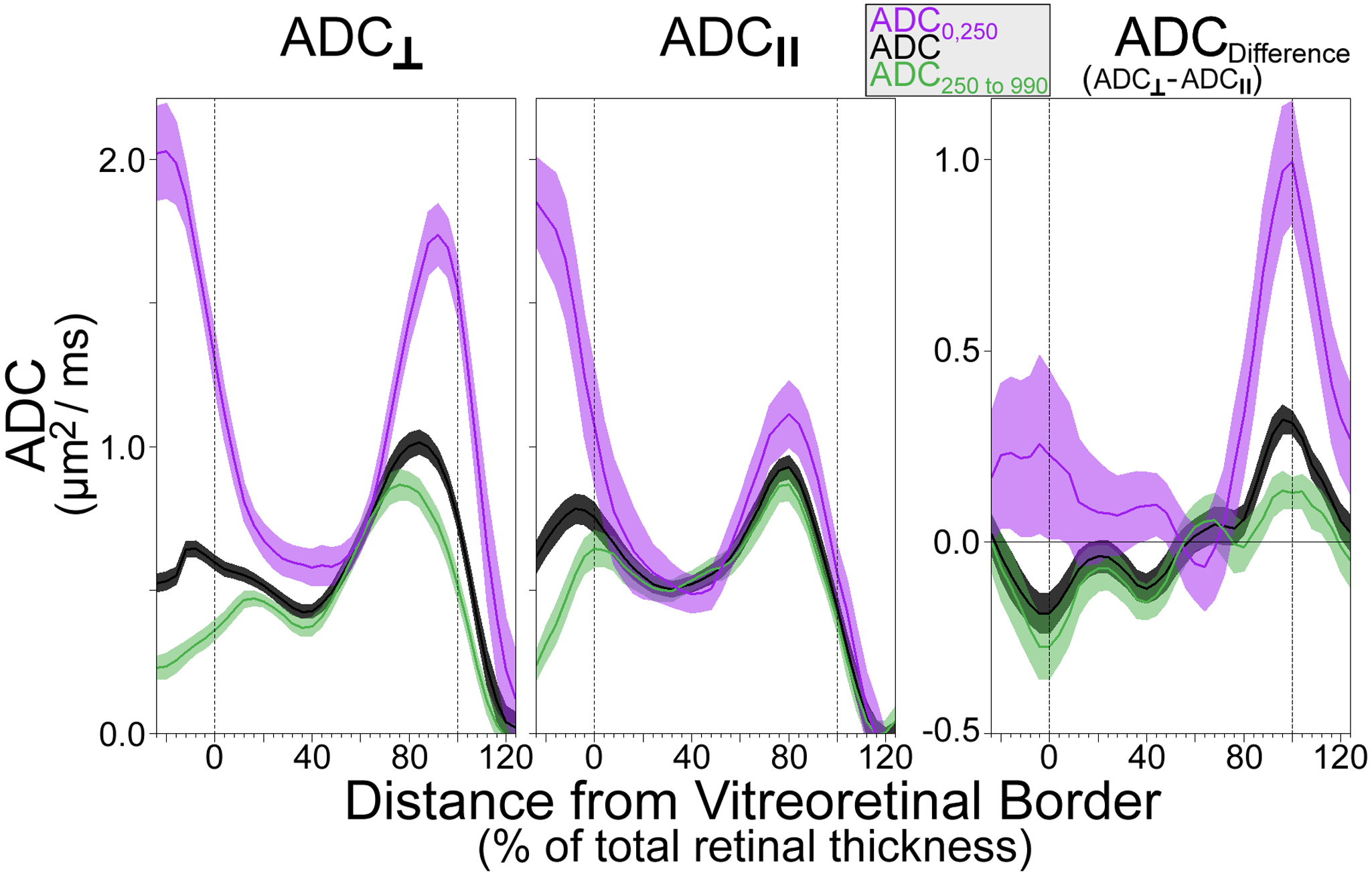

Supplement: Figure S2 — (TIF) [file pone.0029619.s002.tif]

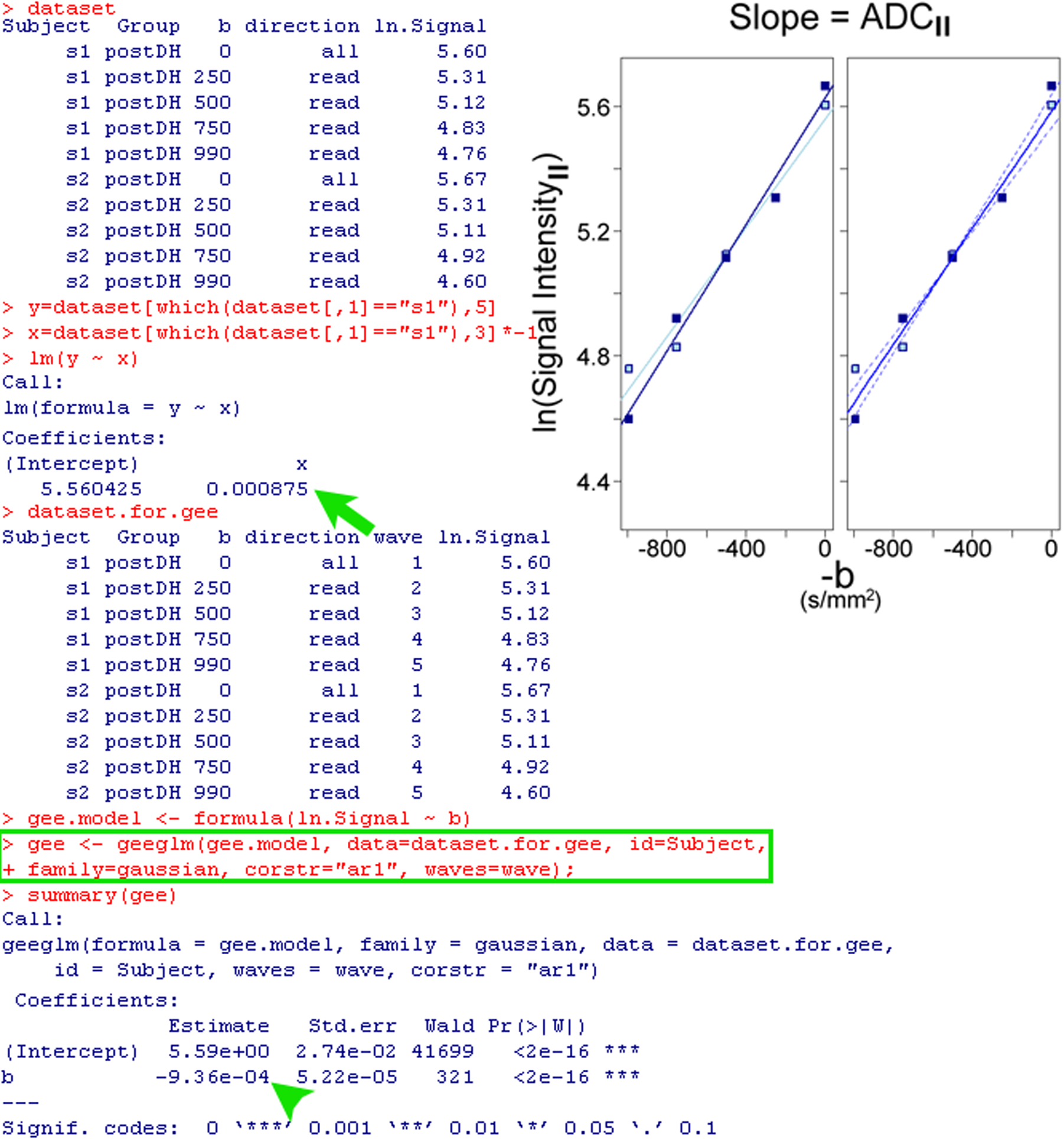

Supplement: Figure S3 — (TIF) [file pone.0029619.s003.tif]

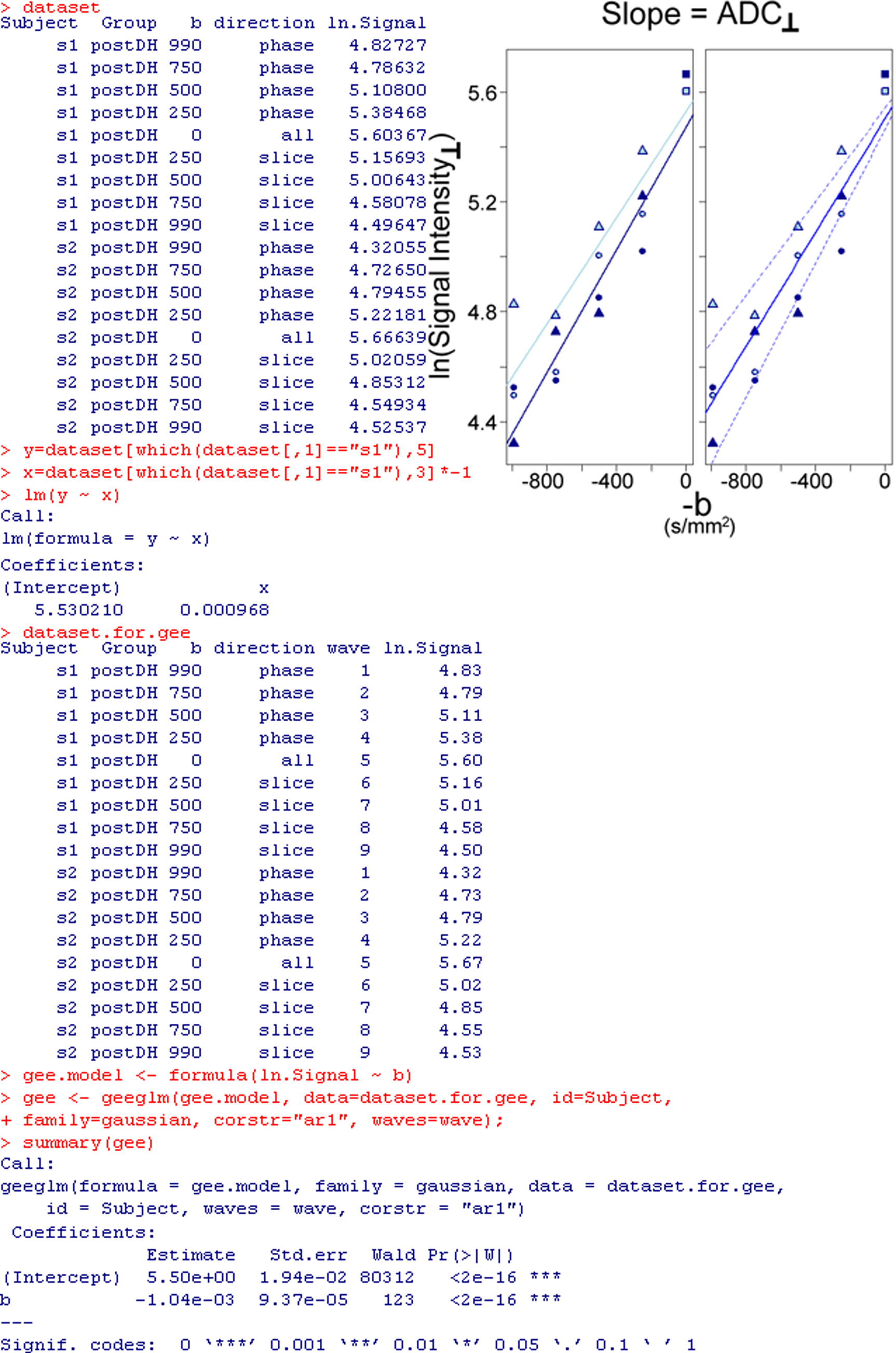

Supplement: Figure S4 — (TIF) [file pone.0029619.s004.tif]

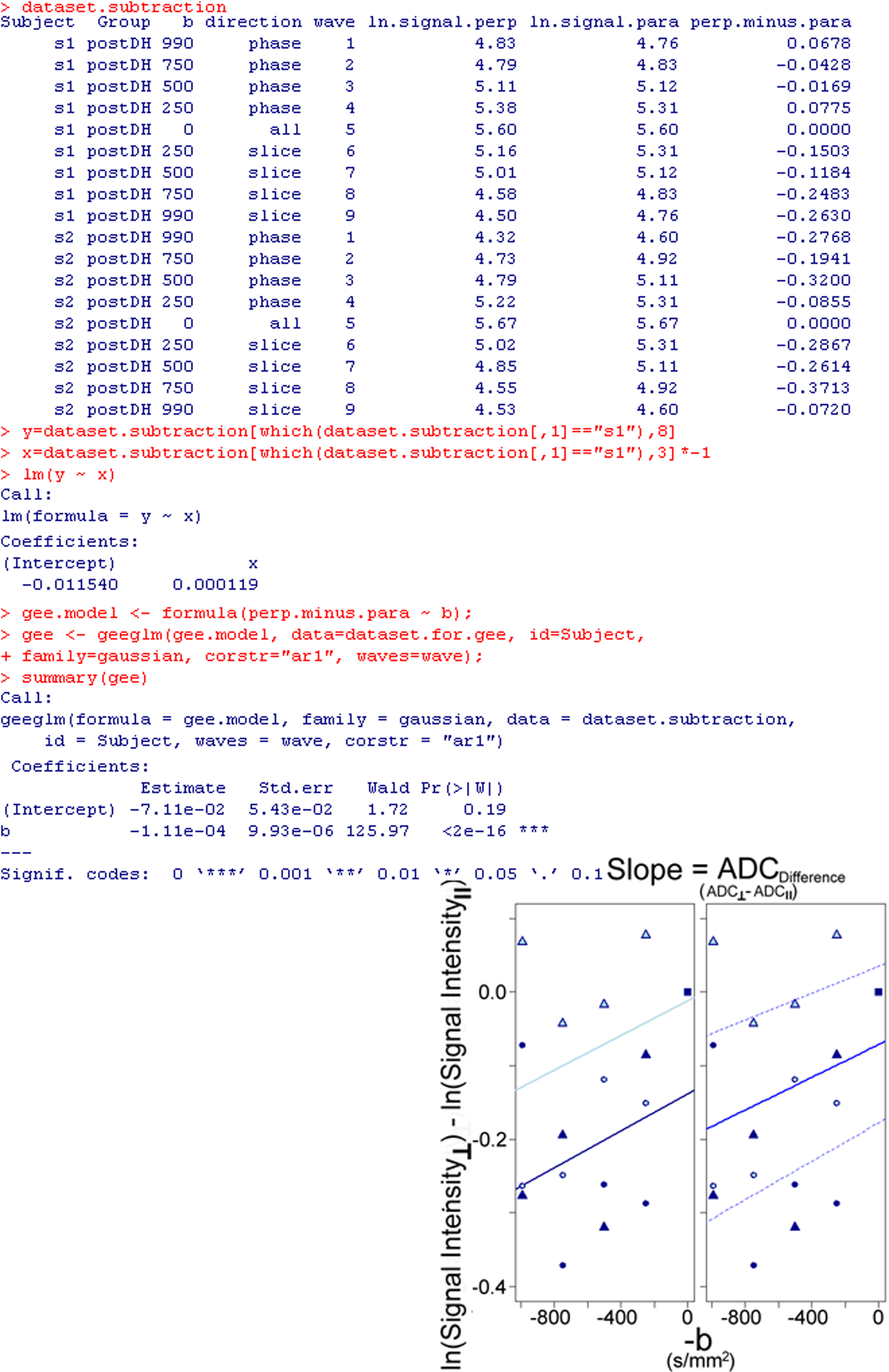

Supplement: Figure S5 — (TIF) [file pone.0029619.s005.tif]

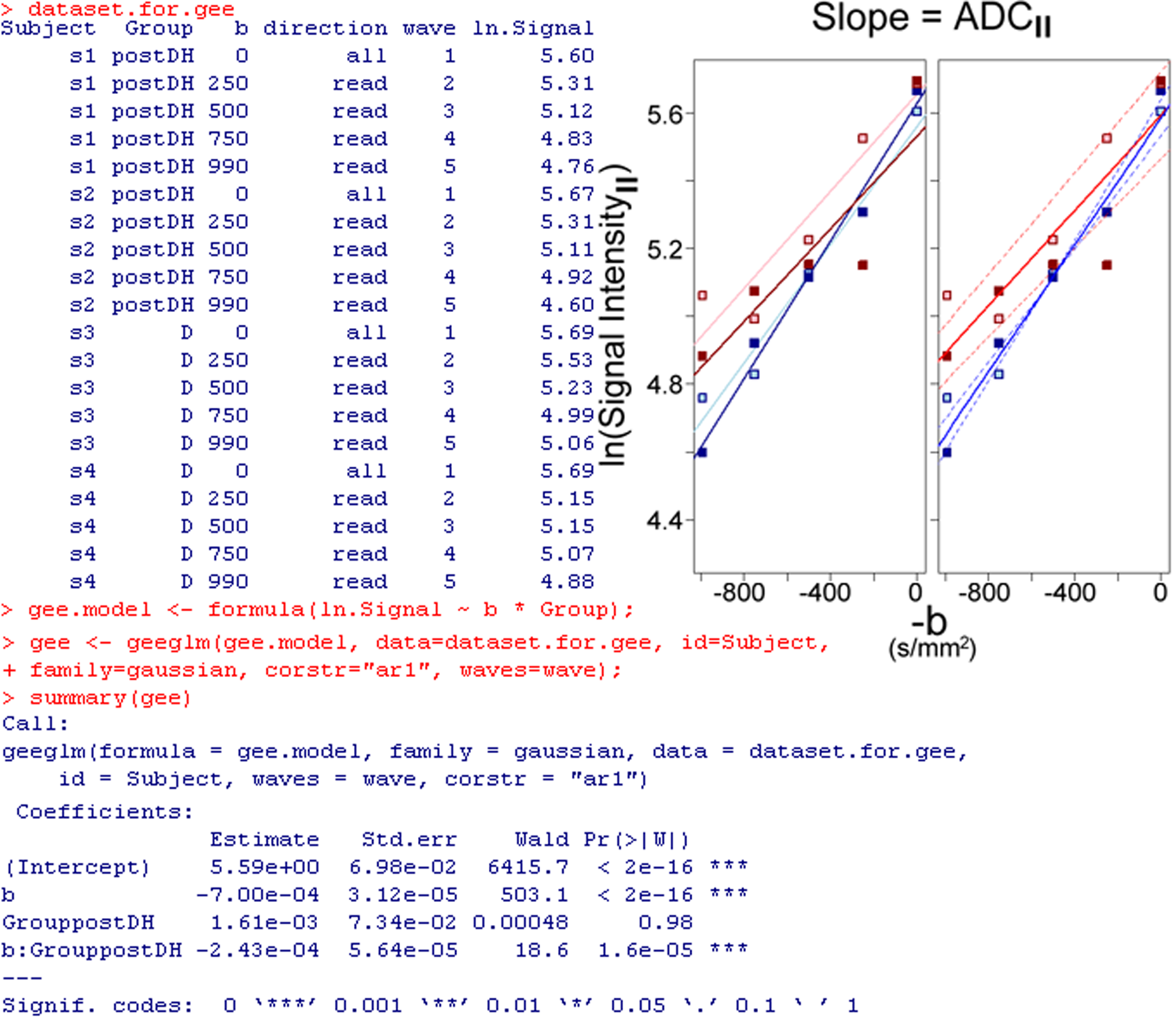

Supplement: Figure S6 — (TIF) [file pone.0029619.s006.tif]

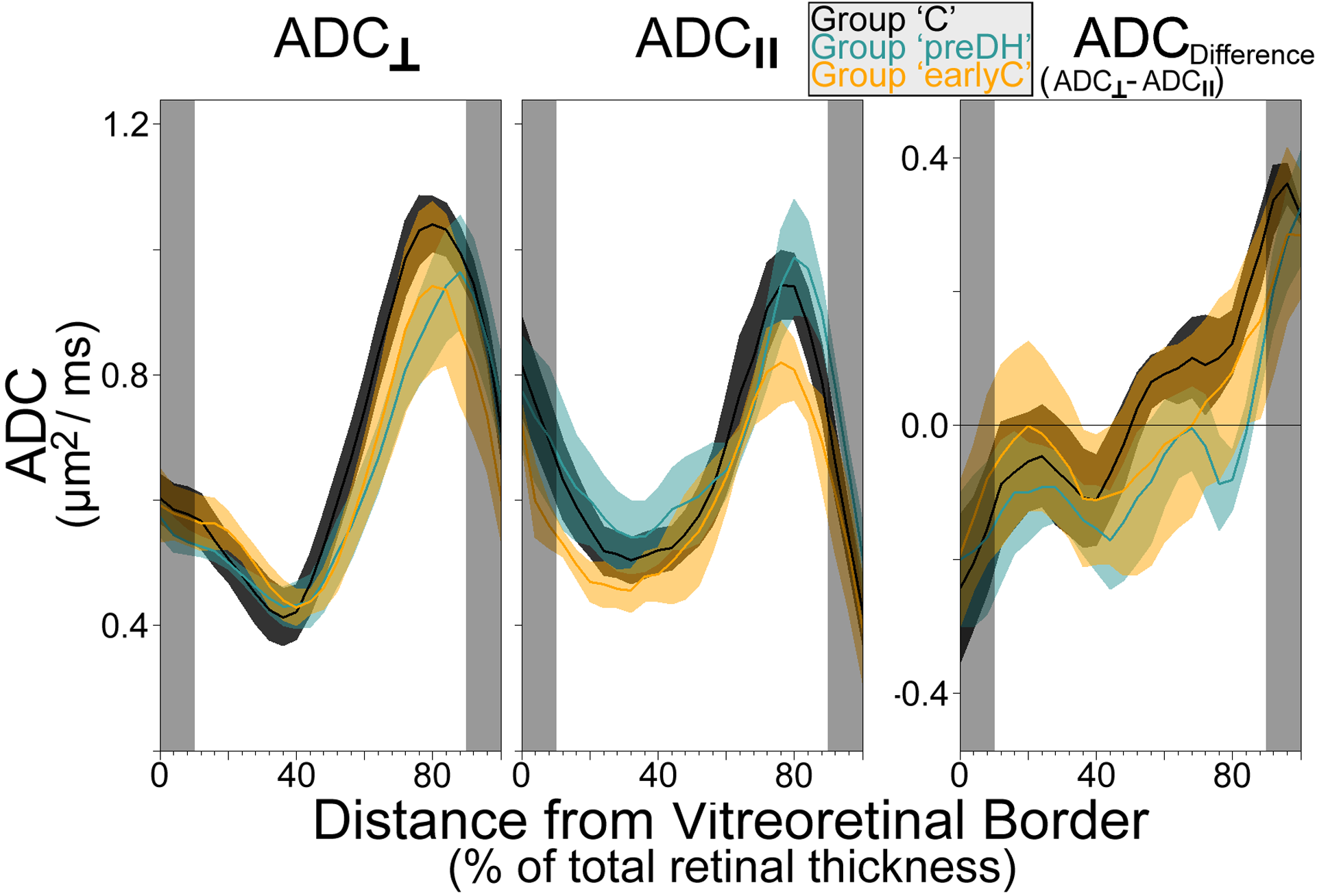

Supplement: Figure S7 — (TIF) [file pone.0029619.s007.tif]
