# Supplementary material for: Evidence for Diffuse Central Retinal Edema In Vivo in Diabetic Male Sprague Dawley Rats
Source: PLoS One. 2012 Jan 11;7(1):e29619. doi: 10.1371/journal.pone.0029619 (PMC3256169; doi:10.1371/journal.pone.0029619)
Supplement: Appendix S1 — (DOCX) [file pone.0029619.s008.docx]

**Appendix S1**

In this appendix we detail how structural and diffusion images were co-registered, ADC was calculated and group comparisons performed, how confidence in the registration process was ensured, and provide evidence demonstrating reproducibility of the diffusion data.

*Registration of Structural and Diffusion Images*

Diffusion images can be somewhat difficult to register to each other and to structural images because signal intensities are greatly reduced at high b-values, and ADC patterns change depending on the local microstructure and flow environments. We therefore developed an algorithmic approach to maximize the quality and reproducibility of registration. In all cases, registration of diffusion-weighted to structural images was performed after linearization of retinal data, which is described in the methods section of the main text and elsewhere [1,2]. For each image, a profile of signal intensity as a function of distance from the vitreoretinal border was obtained from the average superior and inferior central retina. As detailed in Figure S1 and its legend, registration is based on the tissue borders of the signal intensity profiles; alignment was visually confirmed in each case. While reasonable alignment could be achieved using manual registration, the above automated approach provided a small but noticeable improvement in final co-registration and required less time to perform than the manual approach (data not shown).

*Confidence in the Registration Process*

For the purpose of quantitatively evaluating alignment of ADC to structural data, we considered data spanning from -24% thickness (i.e., in the vitreous) to 124% thickness (i.e., at or near the sclera), from the combined control data of the preDH, earlyC, and C groups. To evaluate the alignment of ADC curves to the vitreoretinal border – a landmark based on structural images and located at 0% thickness – we look at the sharp contrast between the nearly unrestricted water movement in the vitreous (high ADC), and the highly restricted movement (low ADC) of the retina. Rapid diffusion in the vitreous causes large signal loss with even modest b-values. With the present image acquisition parameters, vitreous signal intensity remains slightly higher than that of background noise when b = 250 s/mm^2^ (ratio of signal to noise ~1.1 regardless of diffusion direction); for b ≥ 500 s/mm^2^, the vitreous is at noise level. Thus, the ADC-derived vitreoretinal border is found with ADC values calculated using only b = 0 and b = 250 s/mm^2^ (i.e., ADC_║,0,250_ and ADC_┴,0,250_). For completeness, we show these values alongside ADCs calculated using only the four b-values ≥ 250 s/mm^2^ (ADC_║,250 to 990_ and ADC_┴,250 to 990_) and, also, using all five b-values (ADC_║_ and ADC_┴_) in Figure S2. We find that the vitreoretinal border calculated with ADC_0,250_ (at ~ 0% thickness; Table S1) falls close to the vitreoretinal border calculated based on structural data (at 0% thickness), indicating that ADC and structural data are well-aligned using the algorithmic approach.

Next, we illustrate how the alignment of ADC curves to the retina/choroid border – a landmark based on structural images and placed at 100% thickness – was verified. We take advantage of the fact that, in contrast to the nearly isotropic movement of water in the vitreous, movement of choroidal water is relatively directionally restricted with the bulk of the perfusion being parallel to the surface of the eye (i.e., perpendicular to the optic nerve). In addition, fast flowing blood is expected to produce high ADC values but primarily at low b values (e.g., 250 s/mm^2^), again because high mobility water signal-to-noise rapidly decreases at b values > 250 s/mm^2^ [3]. Thus, choroid ADC_0,250_ values are expected to depend heavily on the direction of the diffusion weighting such that ADC_┴,0,250_ > ADC_║,0, 250_. These anticipated patterns were indeed found as illustrated in Figure S2. To better highlight the difference due to choroid flow, for each subject, we subtracted the ADC_║,0,250_ profile from the ADC_┴,0,250_ profile (Figure S2). High values on this ADC_difference,0,250_ profile correspond to areas of both highly direction-dependent and highly unrestricted water movement – areas that match *a priori* expectations for the location of the choroidal circulation [4,5]. As an additional check, we next examined the thickness of this presumptive choroid. In an earlier report, we found that Sprague Dawley rats had a choroidal thickness of ~65 μm *in vivo* [4]. That value was slightly larger than the ~50 μm measured with histology due to lack of perfusion with death and, at least in part, to partial-volume averaging being exacerbated by curvature of the eye into/out of the image plane. From prior


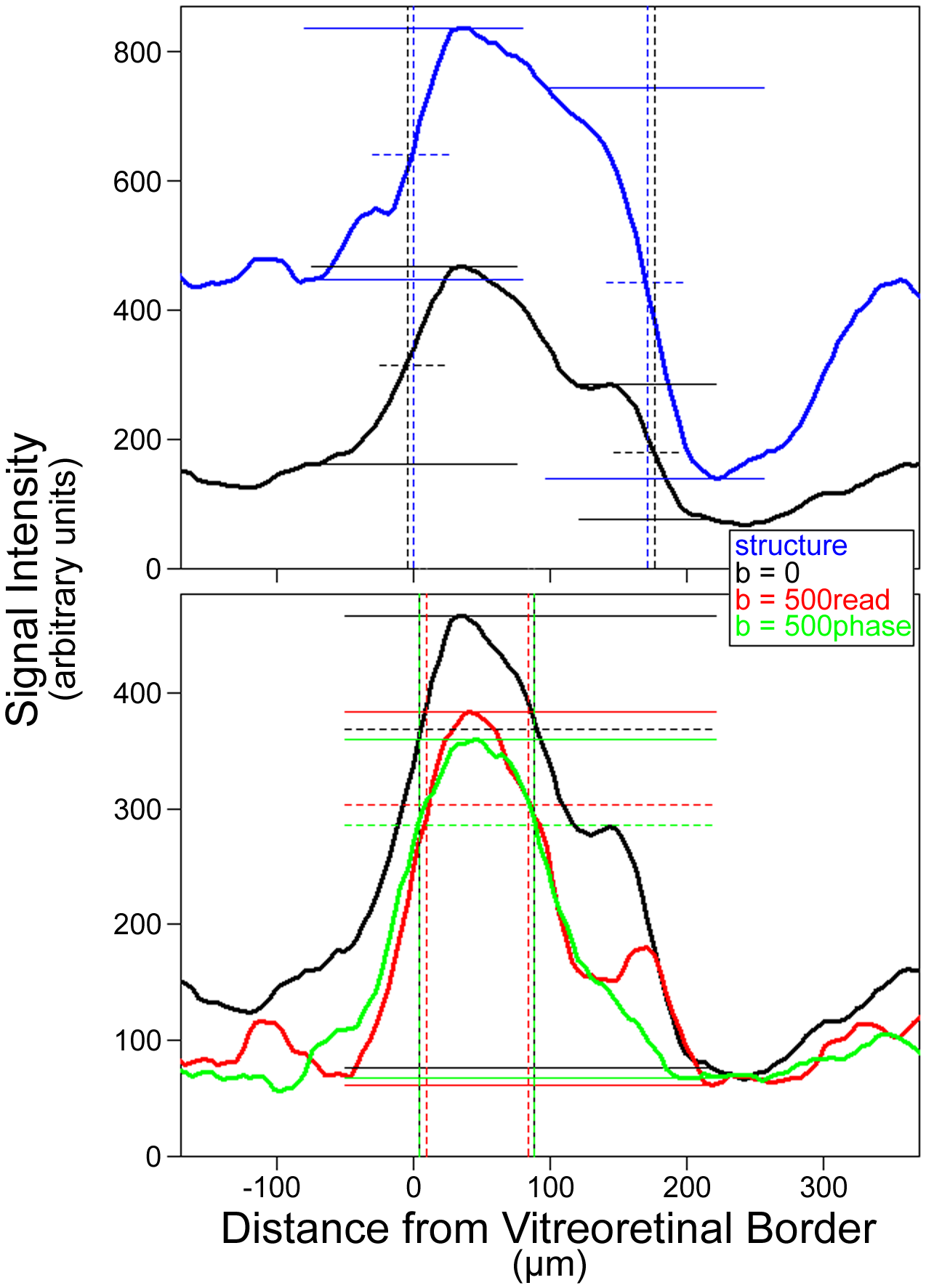


**Figure S1:** *Illustration of registration steps.* These selected signal intensity profiles were calculated from the same control (preDH) image set featured in the main text Figures 3 and 4: (a) in blue, from the averaged pair of structural images, (b) in black, from the averaged set of four acquisitions using the diffusion sequence but with b set to 0, (c) in red, from the image weighted for diffusion parallel to the optic nerve (i.e., up/down in main text Figure 3 and Figure4) with b set to 500 s/mm^2^, and (d) in green, from the image weighted for diffusion in one of the directions perpendicular to the optic nerve (in the phase-encode direction; left/right in Figure 3 and Figure 4, main text) with b set to 500 s/mm^2^. *Top:* For these profiles, localization of the vitreoretinal and retina-choroid borders is accomplished with a previously validated half-height method [1,5]: Solid, color-coded, horizontal lines mark the local minima and maxima of each profile while their horizontal extent indicates the span evaluated when locating a border. In our experience with structural data, checking within 80 μm of provisional borders yields reliable results (data not shown). Slightly narrower spans (within 50 μm) are used with the “b = 0” data in part to avoid inclusion of higher-intensity inner-retinal data when calculating the outer retinal border. Dashed horizontal lines in the top panel of Figure S1 mark the half-height signal intensity. We label the points where the profiles cross their half-height intensities as the vitreoretinal and retina-choroid borders (vertical dashed lines). As shown there, the “b = 0” profile is centered on the structural profile such that the calculated inner and outer borders of the “b = 0” profile are equidistant from the respective inner and outer structural borders. *Bottom:* Once the “b = 0” profile was aligned to the structural profile, all diffusion-weighted images were aligned to the “b = 0” image. Because images collected with b > 0 show diffusion direction-dependent signal losses – the persistence of an outer-retinal ‘bump’ near 180 μm in b_500read_ but absence in b_500phase_ is typical – straightforward application of the half-height method is problematic, and preliminary attempts produced occasional large registration errors (not shown). Instead, we centered the ‘bump’ of high signal present in the inner retina of all diffusion data collected with b > 0 on the “b = 0” profile: Inner and outer borders of this profile contour (vertical dashed lines) were demarcated by where the profile crosses the three-quarter height (horizontal dashed lines; maximum * 3 / 4 + minimum * 1 / 4), then set to be equidistant from the respective inner and outer demarcations of the “b = 0” profile. As in the top panel, solid horizontal lines mark the local minima and maxima of each profile while their horizontal extent indicates the span evaluated when locating borders. Note that in the images used to calculate these profiles, signal intensity of background noise was roughly 90 units.


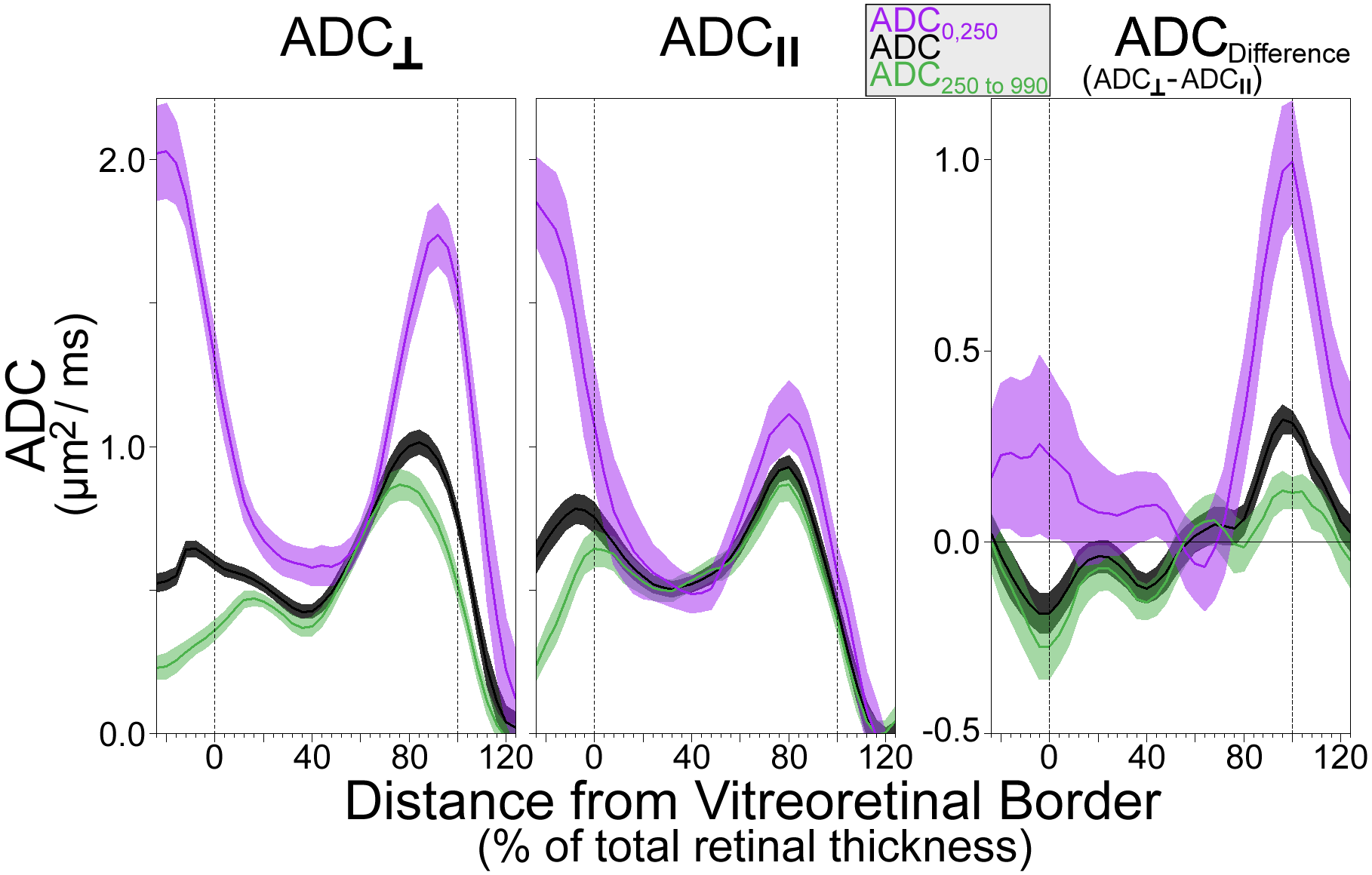


**Figure S2:** *Comparison of ADC profiles using different combinations of b values.* Mean (± s.e.m.) profiles of ADC_0,250_ (purple), ADC_250 to 990_ (green) and ADC calculated with all five b-values (black) shown for the average of two diffusion directions mutually-perpendicular to the optic nerve (left panel; ADC_┴_), the direction parallel to the optic nerve (center panel; ADC_║_), and the difference between the two directions (right panel). To calculate the ADC-derived location of the vitreoretinal border, we used the half-height approach with the local maximum (calculated from -24% to 0% thickness), and the local minimum (from 4% to 44% thickness) of each subject’s ADC_║,0,250_ and ADC_┴,0,250_ profile (see Table S1). A similar calculation near the retina/choroid border showed that local maximums for ADC_║,0,250_ and ADC_┴,0,250_ differed significantly, though local minimums were similar (see Table S1). This expected direction-dependence was used to measure the location and width of the choroid: In each subject’s ADC_difference,0,250_ profile, the half-height method was applied using the local minima (from 56 to 96% thickness) and maxima (56 to 124% thickness) to find the ADC-derived retina/choroid border. The outer border of the choroid was defined as follows: Partial-volume averaging with the sclera, which has very low signal intensities in all images, causes ADC values to progressively become low but highly variable towards 124% thickness. To provide a rough approximation of the border, however, we use the same mid-value that defines the ADC-derived retina/choroid border to find the ADC-derived choroid/sclera border in 11 of the 17 subjects. For the remaining six subjects, for whom the ADC_difference,0,250_ profile remained above the assigned mid-value through 124% thickness, we assigned a value of 128% thickness (one 4% thickness increment past the end of the profile) to the choroid/sclera border. Other possible approaches (e.g. linear extrapolation of ADC_difference,0,250_ values, not shown) suggested assigning a slightly higher thickness value to those subjects, but this yielded only minor changes to the mean values (e.g. assigning 134% places the group mean border at ~117% thickness, with choroid spanning an average of ~58 μm). Considering the profiles calculated with other b-values, we note that the ADC_difference,250 to 990_ profile deviates substantially from the ADC_difference,0,250_ profile as discussed above. For instance, ADC_difference,250 to 990_ is significantly less than 0 from 36% to 44% thickness (q < 0.05; n = 17), indicating greater water mobility parallel to the optic nerve in that region. A similar direction-dependence was noted in mice, but over a broader portion of the retina [8]. When the direction-dependence of diffusion is assessed with all five b-values, the resulting profile shared features present in both ADC_difference,250 to 990_ and ADC_difference,0,250_ profiles: Significant direction dependence (i.e., ADC_difference_ significantly different from 0; q < 0.05) was found with ADC_┴_ < ADC_║_ from -12% through 8% thickness and 36% through 44% thickness, while ADC_┴_ > ADC_║_ from 84% through 116% thickness.

**Table S1:Summary of ADC Local Maximums and Minimums**

|  | **Vitreoretinal Border** | | | **Retina/Choroid Border** | | | **Choroid/Sclera Border** |
| --- | --- | --- | --- | --- | --- | --- | --- |
|  | Local Max.  (vitreous)  (μm^2^ / ms) | Local Min.  (retina)  (μm^2^ / ms) | Location  (% thickness) | Local Max.  (choroid)  (μm^2^ / ms) | Local Min.  (retina)  (μm^2^ / ms) | Location  (% thickness) | Location (% thickness) |
| ADC_┴,0,250_ | 2.20 ± 0.14^†^ | 0.49 ± 0.06 | -1.4 ± 1.8 | 1.59 ± 0.10* | 0.61 ± 0.05 | - | - |
| ADC_║,0,250_ | 2.09 ± 0.15^†^ | 0.32 ± 0.12 | 1.0 ± 3.0 | 0.62 ± 0.10 | 0.47 ± 0.08 | - | - |
| ADC_┴,0,250_ - ADC_║,0,250_ | - | - | - | 1.32 ± 0.11 | -0.25 ± 0.10 | 84.9 ± 2.8 | 114.7 ± 2.9 |

*Significantly different (p < 0.05) from ADC_║,0,250_ value in same column.

^†^ Consistent with the literature value for rat vitreous, 2.3 ± 0.4 μm^2^ / ms [7]

studies, we expect that the choroid would partially overlap with the outer edge of the retina (as defined on structural images) by about ≤ half the anticipated 65 μm [4,5]. Given that the mean retinal thickness of the present control rats was 182 ± 4 μm; n = 17, the choroid is expected to have a span of (65 μm / 182 μm ≈) 36% thickness. We also found that the ADC-derived retina/choroid and choroid/sclera borders were respectively located at ~85 and ~115% thickness (see Figure S2 legend for details), and the choroid covers a span of 29.8 ± 2.7% thickness (roughly 54 ± 5 μm) which is centered at the structurally-defined 100% thickness border. Thus, the present positioning and thickness estimate for the presumptive choroid is in agreement with expectations [4,6], and provides additional support for the accuracy of the objective alignment procedure described herein. Despite the confidence provided by the above considerations regarding alignment, it is nevertheless difficult to decipher, at the borders, the relative contributions of retina and adjacent non-retinal tissue due to partial volume averaging. Thus, we limit the analysis to data well within the borders of the retina (i.e., 12 through 88% thickness) – as defined in structural images (main text; [1]).

*Calculating and Comparing of ADC*

After registration, the signal intensity profiles were resampled and expressed as a function of total retinal thickness (instead of microns). The resampled profiles ranged from -24 to 124% thickness in 4% increments, with the vitreoretinal and retina/choroid borders placed at 0% and 100% thickness, respectively (these borders were defined as described in Figure S1 and its legend). Calculation and comparisons of ADC were then carried out at each increment.

Because only 5 b-values were used in this study, ADC is estimated based on a monoexponential fit of diffusion-dependent changes in signal intensity:

**Eq.1**$\ln(\text{S}_{\text{d ,b}}\text{)}=\text{-b}*\text{ADC}_{\text{d}}+\text{ln(S}_{0}\text{)}$

Where S_d,b_ is the signal intensity in a pixel when diffusion weighting is applied in the d direction with a given b-value, and S_0_ is the pixel signal intensity without diffusion weighting gradients applied (i.e., b = 0). For two or more signal intensities generated by two or more unique b-values, ADC for a single subject is calculated as the fit best-fit slope of Eq.1, found through an ordinary least-squares (OLS) approach. Each subject thereby contributes a single ADC_direction_ value to summary statistics (e.g. mean, standard error), or to comparisons of experimental conditions (e.g. unpaired t-test). Within-subjects comparisons can also be performed (e.g., ADC_║_ to ADC_┴_ with a paired t-test, in which case each subject contributes two values). We used the OLS approach for some calculations in the earlier section which examined each subject’s profile for structural borders.

The OLS approach has an important shortcoming: Since each subject contributes a single value to summary statistics and group comparisons, the within-subject variability – the scatter present when calculating ADC with a best-fit of Equation 1 – is ignored in later testing. Even though high within-subject variability would reduce confidence in the accuracy of an ADC value, and low within-subject variability would improve confidence, the high- and low-confidence cases are treated identically in the OLS approach. The OLS approach will thereby tend to yield false-negative results when within-subject variability is low, and to false-positive results when within-subject variability is high.

Instead, we used a generalized estimating equation (GEE) approach, which accounts for within-subject variability, to perform comparisons in the presence of repeated measures. In this method, instead of, for instance, using five points (one per b-value) to calculate each subject’s ADC_║_, then later averaging slopes to calculate a between-subjects mean and standard error, all points (e.g. forty, if eight subjects are used) are simultaneously fit with a GEE model. Equation 1 is easily adapted for this task, and Figure S3 and legend demonstrate both OLS-based and GEE-based calculations for a specific case (Eq.3, below) in R. The resulting GEE estimate (i.e., the ADC) and standard error account for within- and between- subjects variability.

To illustrate the process of calculating ADC, representative data from two dilutional hyponatremia (postDH) and two diabetic (D) retinas (at 72% thickness) will be presented (Figures S3 – S6). To illustrate the sensitivity of GEE to within-subject variability, both OLS and GEE calculations are provided in cases with low (Figure S3), medium (Figure S4), and high (Figure S5) within-subject variability.

While the GEE estimate and standard error may be used to show that ADC is significantly greater than zero (analogous to a one-sample t-test using OLS-based estimates), this is generally not of interest since tissue ADC is always expected to be nonzero *in vivo*. However, just as a paired t-test is carried out by submitting difference scores to a one-sample t-test, a GEE estimate and standard error based on difference scores allows one to perform a paired comparison of ADCs.

Consider the comparison of postDH to preDH to test for the effects of dilutional hyponatremia on ADC_║_. Two versions of Equation 1 (one for each time point) apply:

**Eq.2**$\ln(\text{S}_{\text{||, b, preDH}}\text{)}=\text{-b}*\text{ADC}_{\text{||, preDH}}+\text{ln(S}_{\text{0, preDH}}\text{)}$

**Eq.3**$\ln(\text{S}_{\text{||, b, postDH}}\text{)}=\text{-b}*\text{ADC}_{\text{||, postDH}}+\text{ln(S}_{\text{0, postDH}}\text{)}$

In the GEE approach, we subtract the two equations and simplify to:

**Eq.4**$(\ln(\text{S}_{\text{||, b, postDH}}\text{)-ln(S}_{\text{||, b, preDH}}))=\text{-b}*(\text{ADC}_{\text{||, postDH}}\text{-ADC}_{\text{||, preDH}})+\text{ln(S}_{\text{0, postDH}}\text{/S}_{\text{0, preDH}}\text{)}$

A GEE model based on Eq.4 is supplied with known b-values, and the difference score from each subject at each b-value (left side of equation), and used to estimate the mean and standard error of the slope, (ADC_║,postDH_ - ADC_║,preDH_), and test the null hypothesis that it is not different than 0 – that dilutional hyponatremia has no effect on ADC_║_.

In Figure S3 and legend, we provide a representative example of the use of R (http://www.r-project.org/) for GEE calculations with Eq.3, to compute the mean and standard errors for diffusion in the one direction parallel to the optic nerve (i.e., ADC_║_) for two postDH retinas. In Figure S4 and legend, the same approach is used for calculating ADC_┴_ which is slightly more complicated since there are two diffusion directions that are mutually-perpendicular to the optic nerve. In Figure S5, the paired comparison of ADC_║_ to ADC_┴_ is shown, which resembles the comparison in Eq.4, above, but has the added complexity of subtracting values from the one parallel direction from each of the two mutually perpendicular directions’ values. Lastly, Figure S6 shows a between-group comparison of ADC_║_ – the retinas of two rats from the diabetic group (“D”) are compared to the retinas of two rats from the postDH group.

*Between-Group Reproducibility of ADC*

Three different groups of control rats (preDH, n = 5; earlyC, n = 4; and C, n = 8) were compared to assess the consistency of the ADC profiles.

In each group, we determined the ADC_┴_ and ADC_║_ profiles and used the above GEE approach to compare them at each point from 12% to 88% thickness. No significant group differences in ADC profiles were noted and the profiles were in reasonable agreement with those reported in control rats (Figure S7; each p > 0.09) (6). As an aside, we also note that no differences in retinal thicknesses between groups were found (p = 0.19; not shown). As noted in the main text, retinal thicknesses are in agreement with previous MRI and OCT results. Together, these considerations strongly support the reproducibility of the present methods for measuring ADC and retinal thickness across the retina.

*Within-Group Reproducibility of ADC*

As noted in the methods section of the primary text, four control rats were used to measure ADC twice per animal: shortly after anesthetizing rats (“earlyC”), then again beginning 4.6 ± 0.3 hr after the start of the first scans, still under urethane anesthesia (“laterC”). To verify the within group reproducibility, regardless of prolonged anesthesia, we compared the ADC_┴_ profiles from the “earlyC” and “laterC” scans using the GEE approach at each point from 12% to 88% thickness. ADC_║_ profiles, as well as difference (ADC_┴_ - ADC_║_) profiles were similarly compared. Retinal thicknesses were also compared. No significant differences were found between the first and second time points. The difference (laterC - earlyC) in retinal


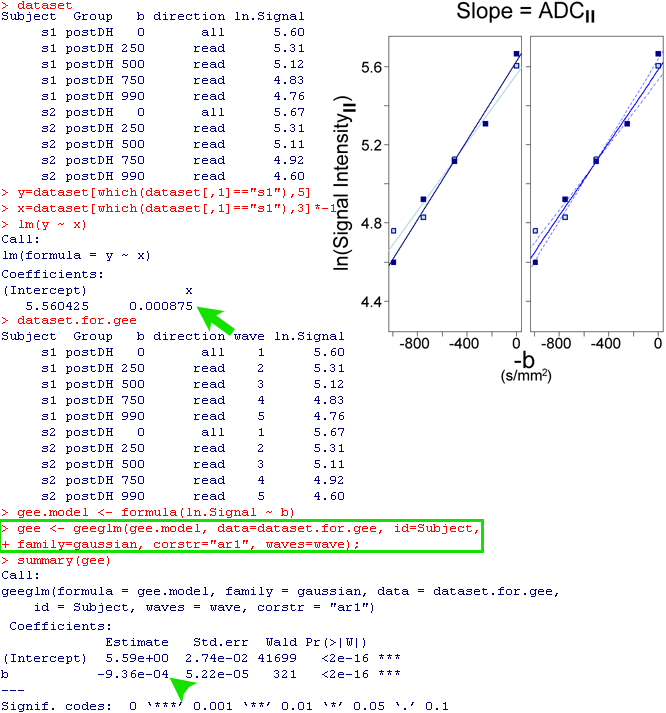


**Figure S3:** *Demonstration of ADC*_║_ *calculation*. Two representative retinal data sets are plotted showing linear change in the natural log signal intensity as a function of b-value (multiplied by -1, so that higher ADC is represented by a steeper slope). As shown in the blue text below ‘>dataset’, the data used here are arranged in rows and columns within ‘dataset’; in the fifth column, the natural log of the measured signal intensity is shown, collected with four levels (250, 500, 750, and 990 s/mm^2^) of diffusion weighting in the ‘read’ direction (i.e., up/down in main text Figures 3 and 4), and data collected without diffusion weighting gradients (i.e., b = 0, which can be applied to ADC calculations for ‘all’ directions), from subjects ‘s1’ and ‘s2’, which belong to the ‘postDH’ group. After setting ‘y’ and ‘x’ equal to, respectively, the log-transformed signal intensities from s1 and their associated b-values (multiplied by -1), the lm() function is used for OLS calculation of s1’s ADC_║_. The coefficient of the best-fit slope (green arrow) would then be multiplied by 10^3^ to match the units used elsewhere in this appendix […]

(here, becoming 0.875 μm^2^ / ms). The same calculation applied to s2 (not shown) yielded an ADC_║_ of 1.018 μm^2^ / ms. The OLS-based mean (± s.e.m.) is therefore 0.95 ± 0.07 μm^2^ / ms. In preparation for ADC_║_ calculation, we create ‘dataset.for.gee’ from ‘dataset’ by adding the ‘wave’ column and values, which are used to specify ‘waves’ in the geeglm() command (green box). In general, GEE statistics can be used to analyze data collected in several “waves”, where values from the nth wave may be somehow related to (or similar to) data from the n+1 and n-1 waves, and progressively less-related to n+2, n-2, n+3, etc. waves; in other words, data that have a correlative structure. Such an autoregressive relationship applies herein (and used by geeglm by setting corstr to “ar1”) because there is a progression of weaker-to-stronger diffusion weighting, as indicated by the sequence of integers in the ‘wave’ column associated with the progressive b-values. The argument ‘family’ is set to ‘gaussian’ because the scatter in our continuous dependent variable (i.e., ln(signal intensity)) has an approximately normal distribution (not shown). For the ‘id’ argument, geeglm() is directed to the ‘Subject’ column of our dataset to bin the repeated measures of each subject: All data points are used at once to calculate a best-fit slope from a single statistical model (‘gee’) that models the correlative structure of the data; these results are summarized at the bottom of this figure. After unit conversion (and multiplication by -1, since, unlike for lm(), b-values were not first multiplied by -1), we find that the GEE model yields an estimate of ADC_║_ of 0.936 ± 0.0522 μm^2^ / ms (green arrowhead) for s1 and s2. The relatively smaller standard error of the GEE vs. OLS-based calculations (i.e., 0.0522 verses 0.07 μm^2^ / ms ) results from the small amount of scatter in each subject’s data (both r^2^ > 0.97). Accounting for intra-individual scatter will often improve the sensitivity of statistical comparisons by reducing false-negative results. Note that, in other cases, the GEE approach can be more conservative than OLS if within-subjects variability is sufficiently high (see Figure S5) thereby reducing the risk of false-positive results. To illustrates this point, we present the results of both OLS and GEE in the following examples (Figures S4, S5, and S6). Note that the R commands displayed in this figure are intended to highlight certain steps of the ADC calculation process, and several useful or essential steps (e.g. use of the library() command to load geepack) are omitted to save space; the entire R script is available upon request of the corresponding author. Data from s1 (light blue) and s2 (dark blue) are plotted with OLS best-fit lines from each subject (left plot), and with the GEE-based best-fit (right plot; solid line) calculated using both s1 and s2 (with 95% confidence interval, dashed lines).

thickness was 6 ± 6 μm; (p = 0.39; paired two-tailed t-test). Profiles of ADC_┴_, ADC_║_, and ADC_┴_ minus ADC_║_, were statistically similar (q > 0.05) and standard errors of (laterC - earlyC) calculations for ADC_┴_ and ADC_║_ were small (< 0.07 and 0.12 μm^2^ / mm respectively) throughout. This consistency also made it possible to find significant effects of the dilutional hyponatremia procedure (see main text) relative to the minimal test/retest differences in controls. These results indicate that prolonged urethane anesthetic does not alter retinal water mobility or thickness, and provide additional confidence in the present analysis protocols and general reproducibility of ADC findings.


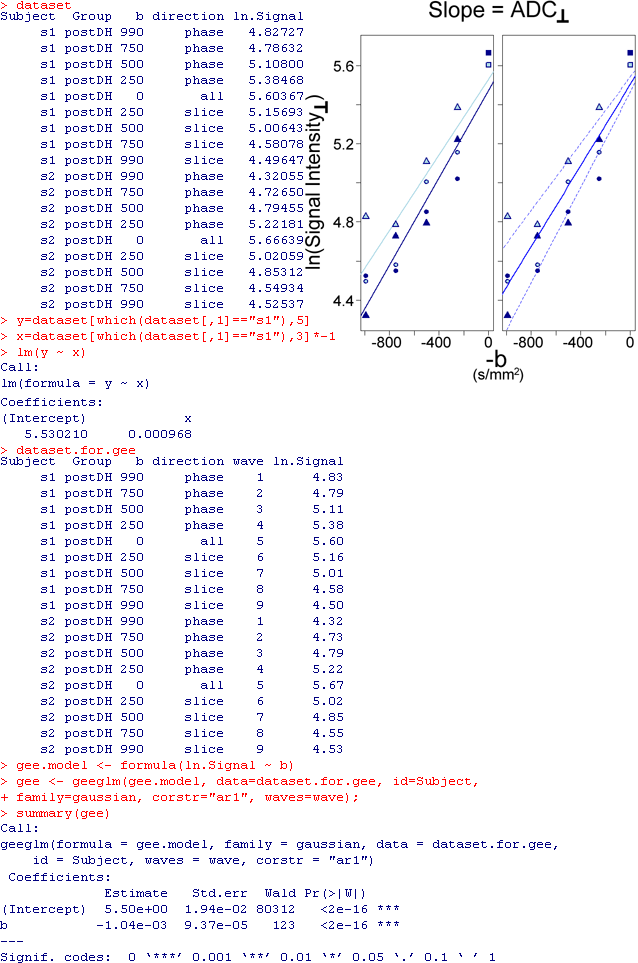


**Figure S4:** *Illustration* *of the calculation of ADC*_┴_. The same two representative postDH retinas used in Figure A3, are analyzed here using plots showing linear change in the natural log signal intensity as a function of b-value (multiplied by -1, so that higher ADC is represented by a steeper slope). The organization and meanings of the R text are detailed in the legend for Figure S3. Note that diffusion-weighted data in each of the two directions mutually-perpendicular to the optic nerve (‘phase’, which is left/right in main text Figures 3 and 4, and ‘slice’, which is into/out of the image plane in those Figures) are used here. No distinction is made between these two directions when calculating ADC, except indirectly in the GEE approach, through use of the ‘wave’ designation in ‘dataset.for.gee’: As noted in the legend for Figure S3, we assume that the measures obtained at progressive diffusion weighting strengths bear an autoregressive relationship to one-another. For instance, were the (natural log of the) signal intensity for b = 750 s/mm^2^ in the phase direction unknown, it would be somewhat predictable based on the value for b = 250 s/mm^2^ in the phase direction, but more predictable based on the value for b = 500 s/mm^2^ in the phase direction. This special relationship between values need not extend to the other direction (here: slice) except through the shared b = 0 value, as reflected in the numbering for the ‘wave’ column. OLS ADC_┴_ results of 1.121 μm^2^ / ms for s2 (not shown) and 0.968 μm^2^ / ms for s1 yield an across-subjects mean (± s.e.m.) ADC_┴_ estimate of 1.04 ± 0.08 μm^2^ / ms. The GEE-based ADC_┴_ is 1.04 ± 0.0937 μm^2^ / ms. Thus, in this […]

case, where scatter is moderate (compare to Figures S3 and S5), the GEE- and OLS-based calculations yield similar estimates and standard errors. As in Figure S3, plots of data from s1 (light blue) and s2 (dark blue) are plotted with OLS best-fit lines from each subject (left plot), and with the GEE-based best-fit (right plot) calculated using both s1 and s2. Circular and triangular points represent data collected with diffusion weighting in, respectively, the slice and phase directions.


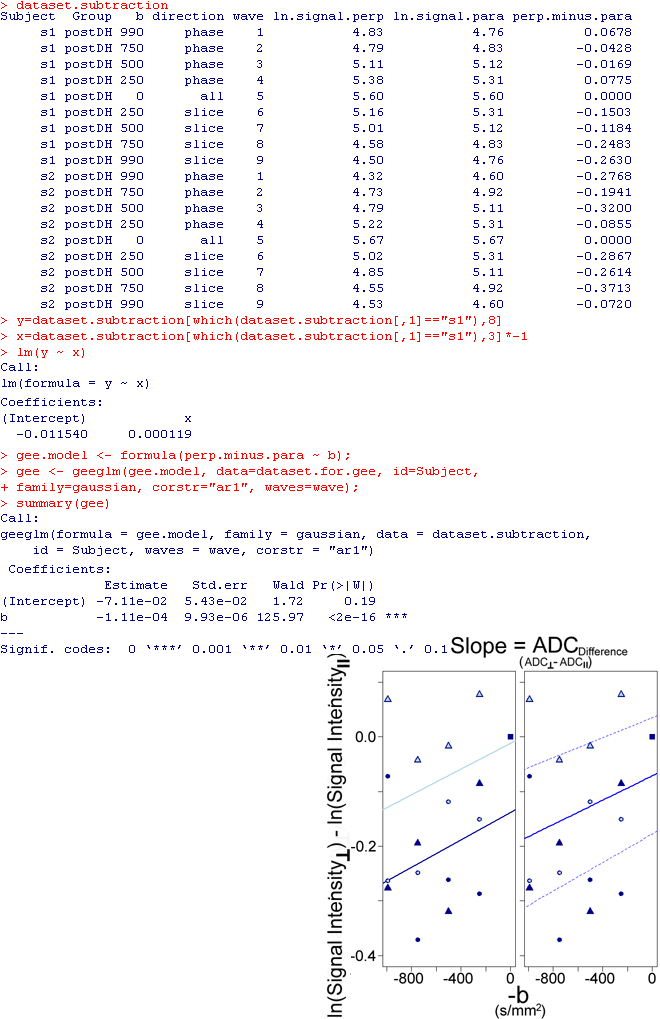


**Figure S5:** *Calculation of ADC*_┴_ *- ADC*_║_. The same two representative postDH retinas featured in Figures S3 and S4 are shown here by plots showing the modest but non-zero relationship between the calculated difference in natural log signal intensity and b-value (multiplied by -1, so that greater differences are represented by a steeper slope). The organization and meanings of the R text are detailed in the legend for Figure S3, and additional information on the relationships between read, phase, and slice directions of diffusion weighting is available in the legend for Figure S4. Note that the seventh column of ‘dataset.subtraction’ is populated with the diffusion data from the parallel (‘read’) direction shown in Figure S3, but with some repetition for each subject, since, for each b-value, it must be subtracted from ‘phase’ data and (in another row) from ‘slice’ data. The eighth column has the results of this subtraction, and – consistent with Equation 4 – is used for OLS calculation of ADC_┴_ - ADC_║_, resulting in 0.125 μm^2^ / ms, for s2 (not shown) and 0.119 μm^2^ / ms for s1. Note that an alternative OLS-based calculation method – direct subtraction of the independently-calculated ADC_║_ (Figure S3 legend) and ADC_┴_ (Figure S4 legend) from each subject – yields similar but non-identical results (s1: (0.968-0.875=) 0.093 μm^2^ / ms; s2: 0.104 μm^2^ / ms). This is because – as was done throughout the rest of this study – data from b = 0 is used only once per subject to calculate ADC_┴_ in Figure S4, and only once per subject in this figure to calculate ADC_┴_ - ADC_║_. If, however, it is used twice per subject – once for the phase, and once for the slice direction – values of 0.998 (s1) and 1.199 μm^2^ / ms (s2) are obtained […]

for ADC_┴_. If the b = 0 data are also used twice in OLS best-fits for ADC_┴_ - ADC_║_ – once for phase minus read, and once for slice minus read – values of 0.123 (s1) and 0.181 (s2) are obtained, in agreement with a direct subtraction of OLS results (e.g. s1: (0.998-0.875=) 0.123 μm^2^ / ms). These considerations highlight a problem with the direct subtraction approach: It requires, without justification, weighting the OLS fit towards the origin of the plot by counting each subject’s single b = 0 datapoint (which necessarily has a y-axis value of 0 in this comparison) twice. This issue motivated the present approach to fitting ADC_┴_ - ADC_║_ data using GEE. Analogous to a paired t-test calculation for ADC_┴_ verses ADC_║_, the mean value for ADC_┴_ - ADC_║_ accompanied by the relatively-small standard error (OLS: 0.122 ± 0.003 μm^2^ / mm; GEE: 0.111 ± 0.00993 μm^2^ / mm) demonstrates that the mean difference is significantly higher than zero, and diffusion is therefore anisotropic and less restricted in the perpendicular than parallel directions to the optic nerve. Note that the standard error calculated here with the separate s1 and s2 OLS fits suggests more confidence in the mean value than is justified given the scatter in each dataset (r^2^ < 0.12 for both s1 and s2), and the larger standard error in the GEE approach makes it appropriately more conservative for testing whether the difference (ADC_┴_ - ADC_║_) is greater than 0. As in Figures S3 and S4, data from s1 (light blue) and s2 (dark blue) are plotted with OLS best-fit lines from each subject (left plot), and with the GEE-based best-fit (right plot) calculated using both s1 and s2.


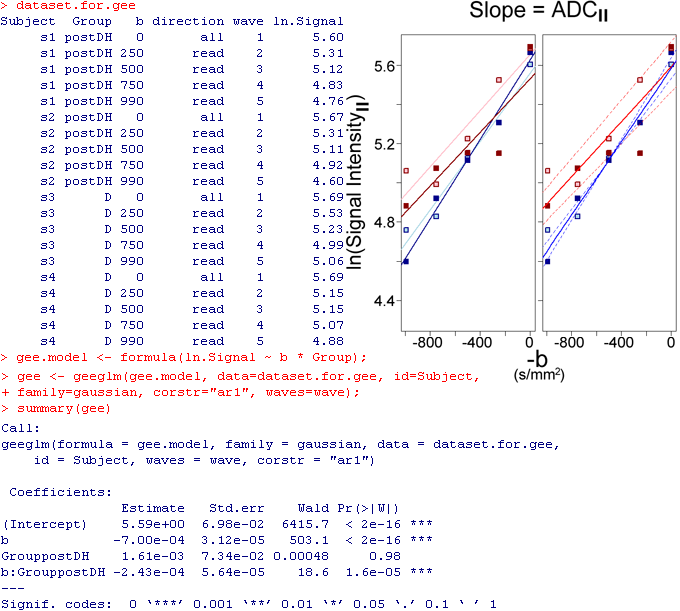


**Figure S6:** *Illustration of between-subjects statistical comparison of retinal ADC*_║_*s.*  The same two postDH rats (also used for Figures S3, S4 and S5) were used to compare the ADC_║_s with that of two representative rats from the diabetic group (‘D’). The organization and meanings of the R text are detailed in the legends for Figures S3 and S4, and (aside from the addition of data from group ‘D’) the plots are identical to those in Figure S3. OLS methods for calculating ADC_║_ using the log-transformed signal intensities and their corresponding b-values were used for all four subjects, but are not pictured here since they are identical to those in Figure S3. Continuing the OLS approach for these four subjects, we found that ADC_║_ in the two D retinas (s3: 0.721 μm^2^ / mm, s4: 0.686 μm^2^ / mm; mean ± s.e.m. of 0.704 ± 0.0175) were not different than in the two postDH retinas (see Figure S3 and legend; 0.95 ± 0.07; p = 0.17 on two-tailed t-test). GEE calculation proceeded as described in previous figure legends, but with ‘gee.model’ including both ‘b’ (the b-value), ‘Group’ (postDH vs. D), and an interaction (denoted by a ‘*’ between the two variables instead of a ‘+’) as predictors of the log-transformed signal intensity. The table below ‘summary(gee)’ gives the results: The row labeled ‘GrouppostDH’, represents a test for global group differences in log-transformed signal intensity (e.g. if slopes for each group were similar, but the D group values were consistently higher than in postDH, independent of the influence of b-value), and is not of interest at present. Here, we are interested in ADC – the relative change in log-transformed signal intensity as a function of b-value. In contrast to its meaning in Figure S5 (where it tested whether the ADC difference was nonzero), the significant finding in row ‘b’ of the summary table trivially means that, across-subjects, b-value has a nonzero influence on signal intensity. We are instead most interested in the ‘b:GrouppostDH’ row, which shows that the Group-by-b-value interaction is significant (p < 0.05) – that the slope (i.e., ADC_║_) differs depending on group, being ~ 0.243 ± 0.0564 μm^2^ / mm higher in the postDH than in the D retina.


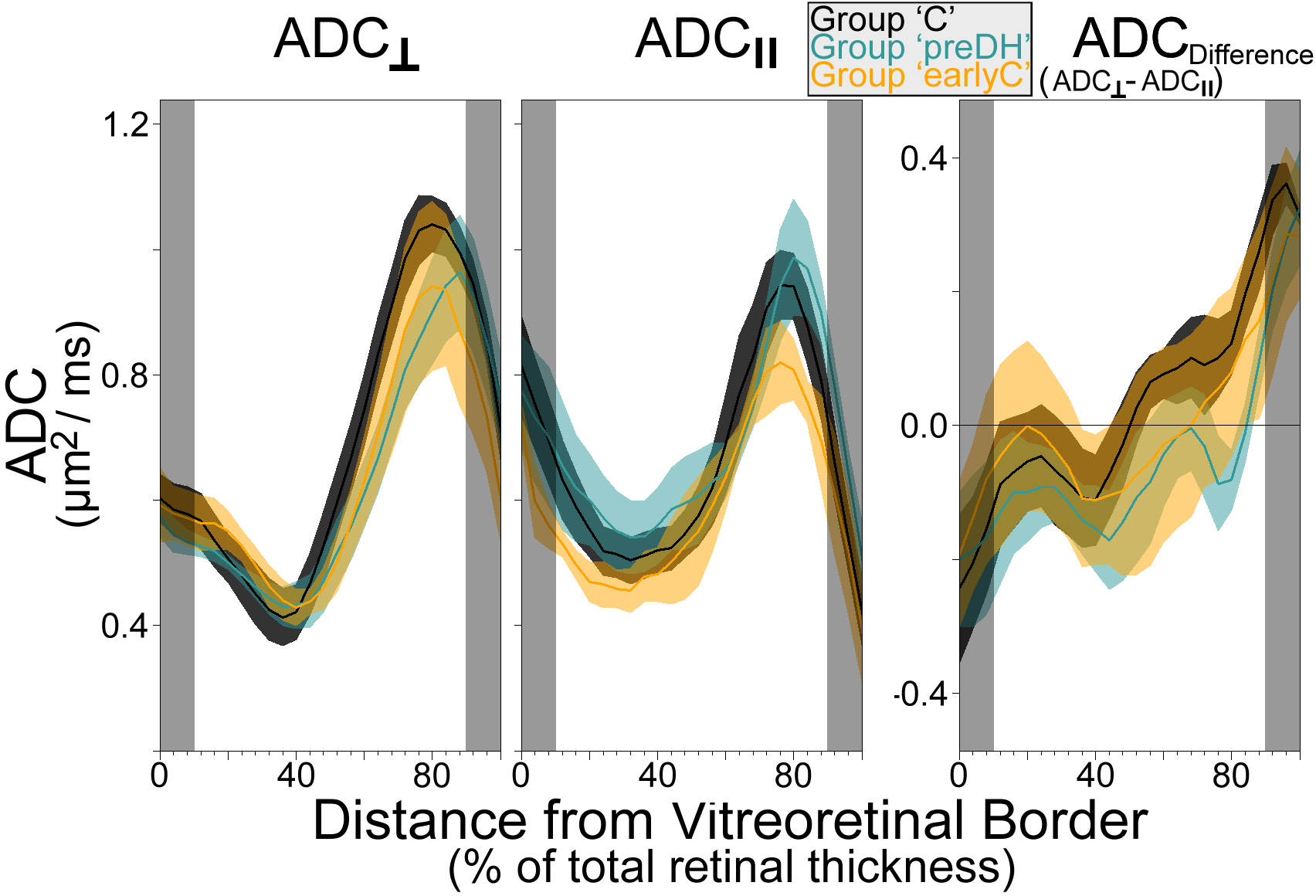


**Figure S7:** *Demonstration of between-group reproducibility.* Control groups’ intraretinal ADC profiles calculated with all five b-values shown for the average of two diffusion directions mutually-perpendicular to the optic nerve (left panel; ADC_┴_) and the direction parallel to the optic nerve (right panel; ADC_║_). Solid lines – black for group C, turquoise for preDH, and orange for earlyC – (with shaded areas) represent the group means (± s.e.m.). Only 12 through 88% thickness regions are analyzed, and vertical shaded areas denote border regions excluded to avoid partial-volume averaging. Groups were statistically similar (P > 0.05) at each %thickness (from 12 through 88% in 4% increments).

**References**

1. Bissig D, Berkowitz BA (2011) Same-session functional assessment of rat retina and brain with manganese-enhanced MRI. NeuroImage 58: 749-760.

2. Berkowitz BA, Roberts R, Bissig D (2010) Light-dependant intraretinal ion regulation by melanopsin in young awake and free moving mice evaluated with manganese-enhanced MRI. Mol Vis 16: 1776-1780.

3. Yacoub E, Uludag K, Ugurbil K, Harel N (2008) Decreases in ADC observed in tissue areas during activation in the cat visual cortex at 9.4 T using high diffusion sensitization. Magn Reson Imaging 26: 889-896.

4. Berkowitz BA, Roberts R, Luan H, Bissig D, Bui BV, et al. (2007) Manganese-enhanced MRI studies of alterations of intraretinal ion demand in models of ocular injury. Invest Ophthalmol Vis Sci 48: 3796-3804.

5. Cheng H, Nair G, Walker TA, Kim MK, Pardue MT, et al. (2006) Structural and functional MRI reveals multiple retinal layers. Proc Natl Acad Sci U S A 103: 17525-17530.

6. Nair G, Tanaka Y, Kim M, Olson DE, Thule PM, et al. (2011) MRI reveals differential regulation of retinal and choroidal blood volumes in rat retina. NeuroImage 54: 1063-1069.

7. Nair G, Shen Q, Duong TQ (2010) Relaxation time constants and apparent diffusion coefficients of rat retina at 7 Tesla. Int J Imaging Syst Technol 20: 126-130.

8. Wang Q, Song SK, Zhang H, Berkowitz BA, Chen S, et al. (2011) Photoreceptor degeneration changes magnetic resonance imaging features in a mouse model of retinitis pigmentosa. Magnetic Resonance in Medicine 65: 1793-1798.
